# Cotrimoxazole Prophylaxis Discontinuation among Antiretroviral-Treated HIV-1-Infected Adults in Kenya: A Randomized Non-inferiority Trial

**DOI:** 10.1371/journal.pmed.1001934

**Published:** 2016-01-05

**Authors:** Christina S. Polyak, Krista Yuhas, Benson Singa, Monica Khaemba, Judd Walson, Barbra A. Richardson, Grace John-Stewart

**Affiliations:** 1 US Military HIV Research Program, Walter Reed Army Institute of Research, Bethesda, Maryland, United States of America; 2 Department of Medicine, University of Washington, Seattle, Washington, United States of America; 3 Department of Global Health, University of Washington, Seattle, Washington, United States of America; 4 Kenya Medical Research Institute, Nairobi, Kenya; 5 Department of Pediatrics, University of Washington, Seattle, Washington, United States of America; 6 Department of Biostatistics, University of Washington, Seattle, Washington, United States of America; 7 Department of Epidemiology, University of Washington, Seattle, Washington, United States of America; St. Vincent's Hospital, AUSTRALIA

## Abstract

**Background:**

Cotrimoxazole (CTX) prophylaxis is recommended by the World Health Organization (WHO) for HIV-1-infected individuals in settings with high infectious disease prevalence. The WHO 2006 guidelines were developed prior to the scale-up of antiretroviral therapy (ART). The threshold for CTX discontinuation following ART is undefined in resource-limited settings.

**Methods and Findings:**

Between 1 February 2012 and 30 September 2013, we conducted an unblinded non-inferiority randomized controlled trial of CTX prophylaxis cessation versus continuation among HIV-1-infected adults on ART for ≥18 mo with CD4 count > 350 cells/mm^3^ in a malaria-endemic region in Kenya. Participants were randomized and followed up at 3-mo intervals for 12 mo. The primary endpoint was a composite of morbidity (malaria, pneumonia, and diarrhea) and mortality. Incidence rate ratios (IRRs) were estimated using Poisson regression.

Among 538 ART-treated adults screened, 500 were enrolled and randomized, 250 per arm. Median age was 40 y, 361 (72%) were women, and 442 (88%) reported insecticide-treated bednet use. Combined morbidity/mortality was significantly higher in the CTX discontinuation arm (IRR = 2.27, 95% CI 1.52–3.38; *p <* 0.001), driven by malaria morbidity. There were 34 cases of malaria, with 33 in the CTX discontinuation arm (IRR = 33.02, 95% CI 4.52–241.02; *p* = 0.001). Diarrhea and pneumonia rates did not differ significantly between arms (IRR = 1.36, 95% CI 0.82–2.27, and IRR = 1.43, 95% CI 0.54–3.75, respectively). Study limitations include a lack of placebo and a lower incidence of morbidity events than expected.

**Conclusions:**

CTX discontinuation among ART-treated, immune-reconstituted adults in a malaria-endemic region resulted in increased incidence of malaria but not pneumonia or diarrhea. Malaria endemicity may be the most relevant factor to consider in the decision to stop CTX after ART-induced immune reconstitution in regions with high infectious disease prevalence. These data support the 2014 WHO CTX guidelines.

**Trial registration:**

ClinicalTrials.gov NCT01425073

## Abstract 2

In a randomized controlled non-inferiority trial, Christina Polyak and colleagues assess whether CTX prophylaxis remains important among HIV+ adults on ART in a malaria-endemic setting.

## Editors' Summary

**Background:**

AIDS has killed 39 million people over the past three decades, and 35 million people (mostly living in resource-limited countries) are currently infected with HIV, the virus that causes AIDS. HIV, which is usually transmitted through unprotected sex with an infected individual, gradually destroys CD4 lymphocytes and other immune system cells. Because destruction of the immune system leaves HIV-infected individuals susceptible to “opportunistic” infections, early in the AIDS epidemic, most HIV-positive individuals died within ten years of infection. In 1996, effective antiretroviral therapy (ART)—drug regimens that stop HIV replicating and allow the immune system to recover its ability to fight infections—became available. For people living in affluent countries, HIV/AIDS became a chronic condition, but because ART was expensive, HIV/AIDS remained fatal in resource-limited countries. In 2003, the international community began to work towards achieving universal access to ART. Now, at least a third of people living with HIV have access to ART, and the global mortality (death) rate from HIV/AIDS is falling.

**Why Was This Study Done?:**

To prevent opportunistic infections, the World Health Organization (WHO) recommends cotrimoxazole (CTX) prophylaxis for HIV-infected individuals. CTX is a combination of two antimicrobial drugs that is active against a range of bacterial, fungal, and parasitic infections; prophylactic antimicrobials are taken to prevent infection. CTX decreases morbidity (illness) and mortality among HIV-infected individuals primarily by reducing the rates of malaria, pneumonia, diarrhea, and severe bacterial infections. The 2006 WHO guidelines for CTX prophylaxis recommend that, in resource-limited countries, HIV-infected patients whose CD4 count is ≤350 cells/mm^3^ blood should take CTX and that, in settings with a high prevalence of HIV infection and limited health infrastructure, all HIV-infected adults should take CTX. However, these guidelines were developed before access to ART was scaled-up, and CTX prophylaxis has risks (for example, drug toxicity) as well as benefits. In this unblinded non-inferiority randomized controlled trial, the researchers investigate the effects of CTX discontinuation among ART-treated HIV-infected adults in Kenya. A randomized controlled trial compares the outcomes of individuals randomly assigned to different treatments; in an unblinded trial, everyone involved in the trial knows who is taking which treatment; a non-inferiority trial investigates whether one treatment is not worse than another treatment.

**What Did the Researchers Do and Find?:**

The researchers enrolled 500 HIV-infected adults living in a malaria-endemic region of Kenya (an area where malaria is always present) who had been treated with ART for ≥18 months, who had a CD4 count of >350 cells/mm^3^, and who were taking CTX. Half the participants continued to take CTX; the remainder stopped taking CTX. After 12 months of follow-up, the combined rate of morbidity (malaria, pneumonia, and diarrhea) and mortality was significantly higher in the CTX discontinuation arm than in the CTX continuation arm (a significant difference is one that is unlikely to have happened by chance). The difference in this primary outcome between the trial arms was driven by malaria morbidity—there were 33 cases of malaria in the CTX discontinuation arm but only one case in the CTX continuation arm. By contrast, the rates of pneumonia and diarrhea did not differ significantly between the trial arms.

**What Do These Findings Mean?:**

Because the difference between the two trial arms in terms of the primary endpoint was greater than the researchers’ predefined non-inferiority limit, these findings suggest that CTX discontinuation is inferior to CTX continuation among ART-treated, immune-reconstituted HIV-infected adults living in a malaria-endemic region. The accuracy of these findings may be affected by the lack of blinding—the clinicians’ care decisions and the patients’ threshold for seeking care may have been influenced by their knowledge of their trial arm assignment. Overall, however, these findings suggest that malaria endemicity should be considered when making decisions about whether to stop CTX after ART-driven immune reconstitution. Indeed, in December 2014, following the release of preliminary data from this trial and the completion of an independent trial on CTX discontinuation, WHO issued supplemental guidelines on CTX prophylaxis. These state that CTX prophylaxis can be discontinued for HIV-infected adults who are clinically stable on ART and have evidence of immune recovery and viral suppression but recommend that CTX prophylaxis be continued regardless of CD4 cell count or HIV/AIDS clinical stage in settings where malaria is endemic and/or severe bacterial infections are common.

**Additional Information:**

This list of resources contains links that can be accessed when viewing the PDF on a device or via the online version of the article at http://dx.doi.org/10.1371/journal.pmed.1001934.

- Information is available from the US National Institute of Allergy and Infectious Diseases on HIV infection and AIDS
- NAM/aidsmap provides basic information about HIV/AIDS, summaries of recent research findings on HIV care and treatment, information on opportunistic infections and on CTX prophylaxis, and personal stories about living with HIV/AIDS
- Information is available from Avert, an international AIDS charity, on many aspects of HIV/AIDS, including information on HIV/AIDS in Kenya, on ART, and on opportunistic infections and CTX prophylaxis: Avert also provides personal stories about living with HIV/AIDS
- WHO provides information on all aspects of HIV/AIDS, including its 2013 guidelines on using ART to treat HIV infection and its December 2014 supplement to these guidelines, which contains updated recommendations on CTX prophylaxis; a factsheet about CTX prophylaxis is also available
- The *2015 World AIDS Day Report* provides up-to-date information about the AIDS epidemic, including progress towards universal access to ART
- More information about this trial is available

## Introduction

Cotrimoxazole (CTX), fixed-dose trimethoprim-sulfamethoxazole, is a low-cost and widely utilized broad spectrum antibiotic used to prevent opportunistic infections in patients with human immunodeficiency virus type 1 (HIV-1). Prophylaxis with CTX has been shown to decrease mortality, morbidity, and hospitalizations among HIV-infected adults [1–8] and children [9], primarily by decreasing rates of malaria, pneumonia, and diarrhea, as well as severe bacterial infections, even in settings with high prevalence of CTX resistance [1–3,10,11].

Recommendations for CTX use in adults with HIV-1 vary by setting. In the United States and Europe, CTX is recommended for HIV-1-infected adults with severe immunosuppression (CD4 count < 200 cells/mm^3^) to prevent *Pneumocystis jiroveci* pneumonia and toxoplasmosis [12–14]. Following antiretroviral therapy (ART), CTX can be discontinued once immune reconstitution is documented (CD4 count > 200 cells/mm^3^) [13,15–17].

In resource-limited countries, the threshold for CTX discontinuation following ART remains undefined. The 2006 World Health Organization (WHO) guidelines recommend CTX prophylaxis for HIV-1-infected patients with a CD4 cell count of ≤350 cells/mm^3^ [14]. For settings with a high prevalence of HIV-1 and limited health infrastructure, WHO guidelines recommend that all HIV-1-infected adults take CTX prophylaxis [14]. However, these guidelines were developed prior to the scale-up of ART. The increased use of ART in sub-Saharan Africa warrants reexamination of this policy. Potential benefits of CTX discontinuation include lower risk of drug toxicity or drug–drug interactions, decreased risk of antimicrobial resistance, decreased costs of care, and lower pill burdens for patients.

At the time of our protocol submission, there were no randomized trial data on the effects of discontinuing CTX following ART in resource-limited settings. However, prior to study initiation, a randomized controlled trial (RCT) conducted in Uganda demonstrated an increased incidence of malaria and diarrhea among ART-treated adults who discontinued CTX compared to those who continued [10]. In this RCT, enrollment criteria did not include a minimum time on ART, making it difficult to draw conclusions regarding immune reconstitution. Moreover, almost all of the malaria cases were not severe and were treated on an outpatient basis [10], and may represent malaria incidence similar to that of HIV-uninfected community members.

We conducted an RCT among adults on ART with evidence of immune recovery (ART for ≥18 mo and CD4 count > 350 cells/mm^3^) to determine whether discontinuation of CTX was non-inferior to continued CTX prophylaxis in decreasing morbidity. Following completion of our RCT, the WHO published supplemental guidelines on the use of CTX [18], informed in part by preliminary data from this RCT.

## Methods

### Trial Design and Participants

We conducted an unblinded, two-arm randomized non-inferiority clinical trial. Two study arms were compared: individuals in the CTX continuation arm continued CTX per Kenyan national guidelines (160 mg trimethoprim/800 mg sulfamethoxazole daily), and individuals in the CTX discontinuation arm discontinued CTX at the time of enrollment. The primary aim of the trial was to determine whether CTX prophylaxis can be discontinued in ART-treated, immune-reconstituted adults without significant harm.

We recruited HIV-infected individuals between February 1, 2012, and August 27, 2012, at the HIV Treatment and Care Clinic, known as the Patient Support Center (PSC), of Homa Bay District Hospital in western Kenya. Individuals were eligible for the study if they were ≥18 y old, HIV seropositive, and taking first-line antiretrovirals (ARVs) with evidence of immune recovery (ARVs for ≥18 mo and CD4 count > 350 cells/mm^3^). Additionally, participants had to be currently taking CTX for HIV and willing to return to the clinic every 3 mo for the 12-mo study follow-up period. We excluded individuals who were pregnant or breastfeeding, who were taking second-line ARVs, or who had a documented allergy to CTX.

### Ethics Statement

The study protocol was approved by the ethical review committee of the Kenya Medical Research Institute and the institutional review boards of the University of Washington and the Walter Reed Army Institute of Research. All participants gave informed consent. Consent was written if literate and fingerprint if illiterate, with the signature of an independent witness. Vestergaard Frandsen donated insecticide-treated bednets and water filters. Alere donated cartridges for the Pima machines used for CD4 count measurements. Data were analyzed by co-author KY.

### Randomization and Masking

The study biostatistician at the University of Washington used a computer-generated blocked randomization sequence to assign participants (1:1) to either the CTX continuation or CTX discontinuation arm. Each participant’s arm allocation was concealed from study investigators, staff, and participants in a randomization envelope until the end of the enrollment visit, when the envelope was opened; participants and study staff were not masked to treatment assignment thereafter.

### Procedures

A history and physical examination was conducted at randomization and at 3-mo intervals during follow-up. At randomization, 6 mo, and 12 mo, specimens were obtained for a complete blood count with CD4 count per Kenyan guidelines [19]. If clinically indicated, HIV-1 RNA viral load was measured, in concordance with PSC protocol. CTX was provided using the PSC infrastructure. Follow-up visits were conducted by a nurse and clinical officer. Data were collected on standardized case report forms at each visit. ART and CTX adherence were assessed by self-report in response to questions about missed doses.

Participants were encouraged to come to the clinic to see study providers for any illness as a sick visit. Sick visits included more intensive workup, with malaria rapid diagnostic test, thick/thin blood smears, and measurement of alanine aminotransferase level and HIV-1 RNA viral load, as clinically indicated and available. Moreover, participants were contacted every 2 wk by study counselors using text messaging or phone calls and asked about recent illness and compliance with study arm protocol. Any participant reporting any symptoms was asked to return to the clinic for evaluation by study staff. To decrease the risk of malaria and diarrhea in this cohort, we provided insecticide-treated bednets and water filters to all participants.

At the completion of the study, participants were transferred back to PSC care providers. Those who had stopped CTX were counseled on restarting CTX again as per Kenyan guidelines [20].

### Endpoints

The primary morbidity/mortality endpoint was a composite of malaria, pneumonia, and diarrhea events and non-trauma mortality events. Malaria was defined as a fever, measured or self-reported, and either a positive rapid diagnostic test or thick smear showing the presence of parasites. Pneumonia was defined as fever, cough, or tachypnea and either a clinically abnormal chest examination or evidence of infiltrate on chest radiograph significant enough to require hospitalization. Diarrhea was defined as a self-report of three or more loose stools in a 24-h period in the past week. An independent clinician (J. Brooks, US Centers for Disease Control and Prevention) who was blind to arm assignment at the time of adjudication evaluated all serious adverse events (SAEs) and designated their likelihood of being research-related. Each event was deemed potentially related to the research if it could have been due to either CTX use or CTX discontinuation.

Prespecified secondary endpoints were 12-mo CD4 change and ART treatment failure. Treatment failure was defined by Kenya National AIDS & STI Control Programme guidelines and was categorized as clinical, immunologic, or virologic [19]. Virologic failure was viral load greater than 1,000 copies/ml. Viral monitoring was not routinely available for most participants given resource constraints at the PSC of the Homa Bay District Hospital. Clinical failure was new WHO stage 3 or 4 illness. Immunologic failure was defined as a decline in CD4 count (falling to or below pre-ART value, falling by 30% or more from treatment peak value, or remaining persistently below 100 cells/mm3 after at least 12 mo of ART) after a period of immune reconstitution. Hospitalization was defined as an overnight admission to a hospital. Adverse events (AEs) were graded 1–5 using standardized US National Institutes of Health Division of AIDS grading criteria [21]. SAEs included all grade 3–5 events and grade 2 events that were serious medical events or those requiring hospitalization [21].

### Statistical Analysis

We estimated the needed sample size for the comparison of interest: the incidence rate ratio (IRR) comparing the rate of morbidity/mortality observed in the CTX discontinuation arm to the rate observed in the CTX continuation arm. We chose a non-inferiority limit of 1.25, thus planning to conclude non-inferiority of CTX discontinuation if the upper bound of the two-sided 90% confidence interval (CI) for the IRR was less than 1.25 [22]. We used morbidity incidence data from a prior study of ART-naïve HIV-infected individuals in Nairobi, Kenya [23], in which serious morbidity (pneumonia, diarrhea, hospitalization) was observed at a rate of 160/100 person-years in ART-naïve HIV-1-infected women at all CD4 levels. We expected to observe a similar incidence, assuming lower morbidity because of ART use but higher morbidity because of residence in western Kenya, which has a higher prevalence of infectious diseases. Assuming an incidence rate of 160/100 person-years and equal incidence rates in each arm, a sample size of 250 individuals per arm (500 person-years total) would result in 800 total morbidity events over the 1-y follow-up, allowing us to rule out an IRR of at least 1.25 with approximately 80% power [24].

For analysis of the primary endpoint, we calculated a combined morbidity/mortality incidence rate for each treatment arm. We used Poisson regression with robust error variance to estimate the IRR, using the natural logarithm of follow-up time as an offset variable. We conducted both intention-to-treat (ITT) and per-protocol (PP) analyses. ITT analyses assigned all follow-up time from a participant to that participant’s original randomization arm. In contrast, PP analyses reflected changes to CTX regimen. Specifically, for participants randomized to discontinue CTX who restarted CTX due to pregnancy, PP analyses excluded person-time following the restart of CTX.

In secondary analyses, we calculated IRRs comparing the CTX discontinuation arm to the continuation arm for each individual component of the morbidity/mortality outcome, as well as for subgroups defined by enrollment CD4 count (≤600 cells/mm^3^, >600 cells/mm^3^) and duration of ART at enrollment (≤5 y, >5 y). We also calculated IRRs for the rate of SAEs and the rate of all grade 2 or higher AEs. To estimate IRRs, we used Poisson regression as described above or exact Poisson regression where appropriate. We compared 12-mo change in CD4 count in the two groups using a linear mixed effects model that included main effects for arm, time, and a time–treatment arm interaction. This model used CD4 data collected at the scheduled enrollment, month 6, and month 12 study visits. We used Cox proportional hazards regression to compare time to ART treatment failure between arms.

An independent data safety and monitoring board (DSMB) was convened four times during the course of the study to review study procedures, participant retention, and safety data by arm and included review of any relevant new data. The DSMB adopted a safety stopping rule based on SAEs deemed potentially research-related (due to either discontinuing CTX or continuing CTX) by the DSMB safety monitor, who would be blind to arm assignment at the time of adjudication. Non-severe (i.e., grade 1 or 2) malaria, pneumonia, and diarrhea events were not included as criteria for early stopping, based on the rationale that rates of non-severe AEs in the CTX discontinuation arm may reflect rates in HIV-uninfected community members and could be offset by non-severe AEs related to CTX use. The interim safety analysis was conducted in accordance with the Lan–DeMets implementation of the O’Brien–Fleming boundary for harm; the alpha spending function was based on an information fraction equal to the interim number of events in all arms divided by the total number of expected events by the conclusion of the study. The interim analysis was conducted at an information fraction of 0.64, and the study stopping criterion was not met.

We used Stata version 12.1 [25] for all statistical analyses.

## Results

**Fig 1:**
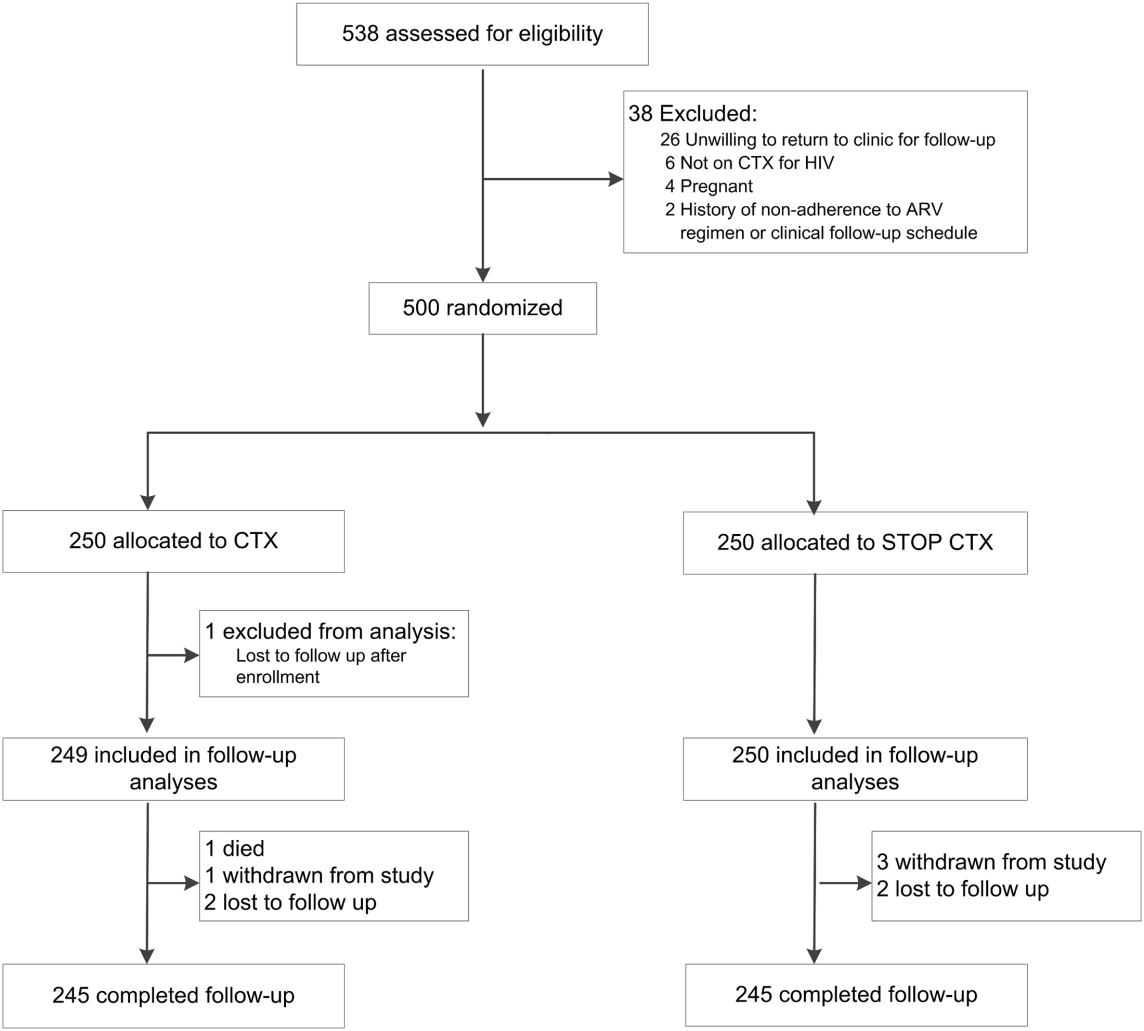
Trial profile.

Five hundred thirty-eight HIV-1-infected adults on ART were screened to meet the target sample size of 500 (Fig 1). Among the 500 randomized participants, 250 were allocated to discontinue CTX, and 250 to continue CTX. Among enrolled participants, 72% were women, 64% were married, 68% had primary school or less education, and 78% had a monthly income of less than 5,000 Kenyan shillings. Median age at enrollment was 40 y, median CD4 count was 595 cells/mm^3^, and median ART duration was 4.5 y. Randomization resulted in baseline characteristics that were generally well-balanced between the two arms (Table 1).

**Table 1:** Characteristics of the study participants at baseline by study arm.

Characteristic	CTX Continuation Arm (*n* = 250)	CTX Discontinuation Arm (*n* = 250)
**Female gender**	184 (73.6%)	177 (70.8%)
**Age, years**	41 (34, 48)	40 (34, 48)
**Marital status**		
Married	156 (62.4%)	165 (66.0%)
Divorced/separated/widowed	90 (36.0%)	77 (30.8%)
Single	4 (1.6%)	8 (3.2%)
**Education (highest completed)**		
Less than primary	15 (6.0%)	18 (7.2%)
Primary school	156 (62.4%)	149 (59.6%)
Secondary school	73 (29.2%)	73 (29.2%)
Vocational school	3 (1.2%)	6 (2.4%)
University	3 (1.2%)	4 (1.6%)
**Estimated monthly income (Kenyan shillings)**		
<5,000	201 (80.4%)	187 (74.8%)
≥5,000	49 (19.6%)	63 (25.2%)
**Number of household residents**	5 (4, 7)	5 (4, 7)
**Number of rooms in residence**	3 (2, 3)	2 (2, 3)
**Water source**		
Piped or well water	135 (54.0%)	133 (53.2%)
Environmental water source	115 (46.0%)	117 (46.8%)
**Toilet type**		
Flush toilet	7 (2.8%)	11 (4.4%)
Pit latrine	196 (78.4%)	202 (80.8%)
Bush	47 (18.8%)	37 (14.8%)
**Bednet use**	220 (88.0%)	222 (88.8%)
**Use of boiled or purified drinking water**	207 (82.8%)	214 (85.6%)
**Hospitalized in past 3 mo**	5 (2.0%)	2 (0.8%)
**Enrollment CD4 count, cells/mm** ^**3**^	598 (487, 695)	591 (515, 719)
**Duration of ART at enrollment, years**	4.5 (3.1, 6.3)	4.5 (3.2, 6.1)

Data are given as *n* (percent) or median (interquartile range).

Study retention was similar in both arms (Fig 1). A total of 490 participants (98%) were retained to the end of scheduled follow-up (the month 12 study visit). A total of five participants were lost to follow-up: three in the CTX continuation arm and two in the CTX discontinuation arm. There were four withdrawals: one in the CTX continuation arm and three in the CTX discontinuation arm. There was one death in the CTX continuation arm.

Scheduled and unscheduled study follow-up visits gave 253.5 person-years of analysis time in the CTX continuation arm and 253.3 person-years of analysis time in the CTX discontinuation arm, among 500 total participants. Participants randomized to the CTX continuation arm self-reported that they took CTX every day in the past week at 90.5% of follow-up visits. By the month 12 study visit, eight of 250 (3.2%) participants randomized to the CTX discontinuation arm had restarted CTX due to pregnancy.

**Fig 2:**
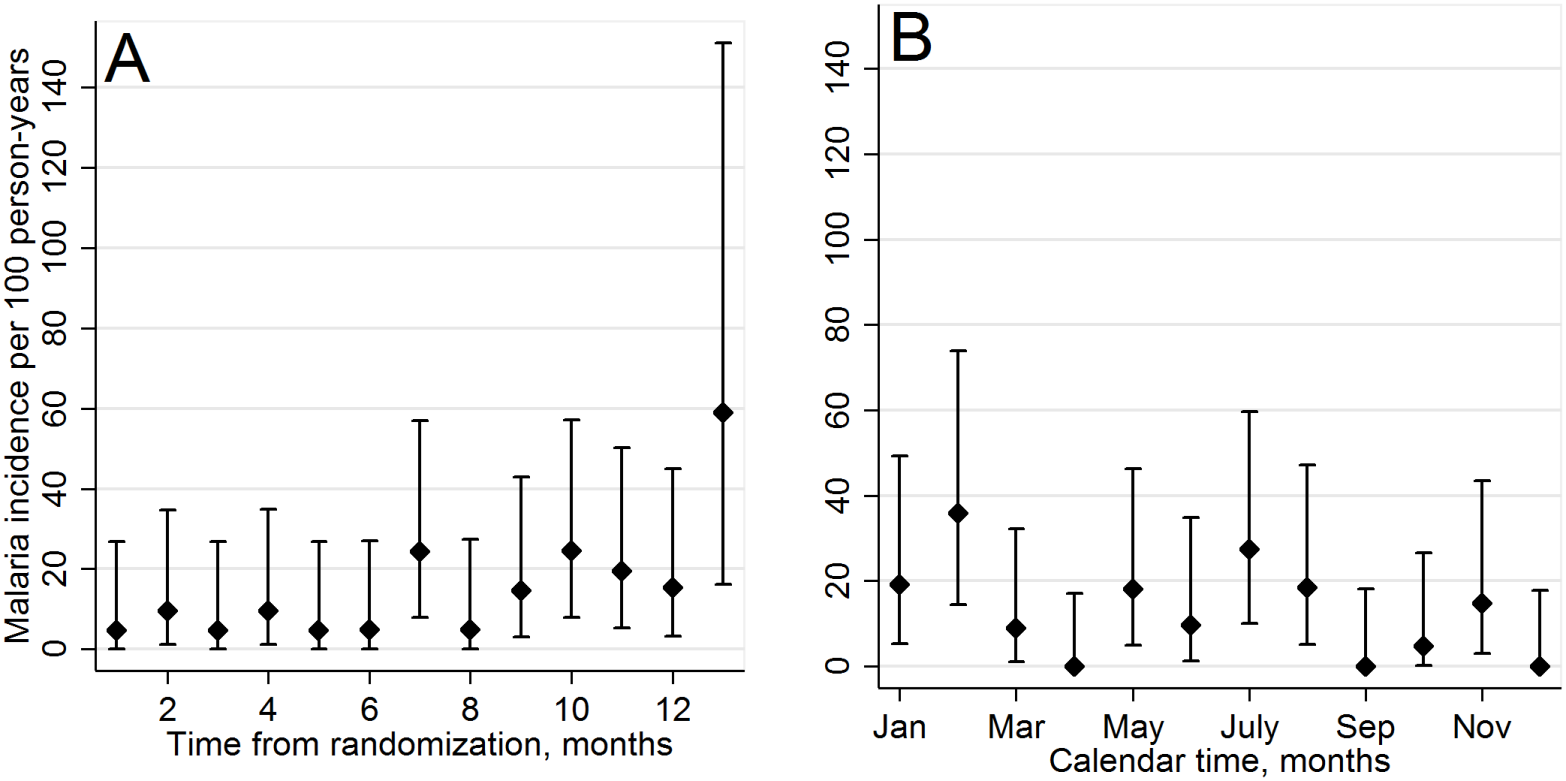
Incidence of malaria cases in CTX discontinuation arm. By study follow-up month (A) and by calendar month (B). Error bars represent the 95% confidence intervals.

For analysis of the primary composite endpoint of morbidity and mortality, a total of 111 morbidity events were observed (Table 2). These consisted of one death, 34 malaria cases (in 32 individuals), 17 pneumonia cases (in 16 individuals), and 59 diarrhea cases (in 50 individuals). In the ITT analysis, the estimated rate of morbidity events was 13.4 per 100 person-years in the CTX continuation arm and 30.4 per 100 person-years in the CTX discontinuation arm, giving an IRR of 2.27 (90% CI 1.62–3.17; 95% CI 1.52–3.38). Given the 90% CI and the highly statistically significant difference in morbidity rates between the two arms (two-sided *p* < 0.001), we could not conclude non-inferiority of CTX discontinuation. The difference by arm was largely driven by the malaria component: the rate of malaria was 0.4 per 100 person-years in the CTX continuation arm and 13.0 per 100 person-years in the CTX discontinuation arm (IRR = 33.02, 95% CI 4.52–241.02). PP results were similar to ITT results, with an IRR of 2.27 (95% CI 1.52–3.39) for the composite endpoint of morbidity and mortality and an IRR of 33.56 (95% CI 4.60–245.01) for malaria. Among individuals in the CTX discontinuation arm, malaria cases occurred throughout the study follow-up period, without evidence of an early “rebound” (Fig 2).

**Table 2:** Morbidity and mortality and adverse event incidence rates by study arm—intention-to-treat analysis.

Outcome or AE Category	Incidence per 100 Person-Years (Number of Cases)		IRR^1^ (95% CI)	*p*-Value^2^
	CTX Continuation Arm (253.5 Person-Years Total)	CTX Discontinuation Arm (253.3 Person-Years Total)		
**Outcomes**				
Combined outcome: malaria, pneumonia, diarrhea, and mortality	13.4 (34)	30.4 (77)	2.27 (1.52, 3.38)	<0.001
Mortality	0.4 (1)	0.0 (0)	1.00 (0.00, 39.02)^3^	0.99
Malaria	0.4 (1)	13.0 (33)	33.02 (4.52, 241.02)	0.001
Pneumonia	2.8 (7)	3.9 (10)	1.43 (0.54, 3.75)	0.47
Diarrhea	9.9 (25)	13.4 (34)	1.36 (0.82, 2.27)	0.24
**AEs**				
SAEs	4.3 (11)	9.1 (23)	2.09 (1.02, 4.27)	0.043
Grade 3 or higher SAEs	3.6 (9)	7.1 (18)	2.00 (0.90, 4.44)	0.088
SAEs deemed potentially related to the research	2.0 (5)	5.1 (13)	2.60 (0.93, 7.28)	0.069
Grade 2 or higher AEs	63.5 (161)	99.5 (252)	1.57 (1.29, 1.91)	<0.001

^1^IRRs estimated using Poisson regression with robust error variance, except where indicated.

^2^For test of null hypothesis: IRR = 1.

^3^Estimated with exact Poisson regression (median unbiased estimate) because of the low rate of mortality events.

In subgroup analyses (Tables 3 and 4), malaria occurred at a statistically significantly higher rate in the CTX discontinuation arm than in the CTX continuation arm across categories of enrollment CD4 count and ART duration at enrollment. For diarrhea, there was effect modification by CD4 count, with an IRR of 3.99 (95% CI 1.51–10.54) in the subgroup with CD4 count ≤ 600 cells/mm^3^ and an IRR of 0.66 (95% CI 0.33–1.31) in the subgroup with CD4 count > 600 cells/mm^3^ (interaction *p*-value = 0.003).

**Table 3:** Subgroup analyses by enrollment CD4 count: comparing CTX discontinuation to CTX continuation arms.

Outcome	Enrollment CD4 Count ≤ 600 cells/mm^3^	Enrollment CD4 Count > 600 cells/mm^3^	*p*-Value for Interaction
Combined outcome: malaria, pneumonia, diarrhea, and mortality	4.33 (2.11, 8.86)	1.46 (0.88, 2.42)	0.015
Mortality	—	—	—
Malaria	18.71 (3.15, +Inf)^1^	19.25 (2.59, 143.38)	—^2^
Pneumonia	1.42 (0.40, 5.04)	1.35 (0.30, 6.01)	0.96
Diarrhea	3.99 (1.51, 10.54)	0.66 (0.33, 1.31)	0.003

Data given as IRR (95% CI). IRRs were estimated using Poisson regression with robust error variance, except where indicated.

^1^Estimated with exact Poisson regression (median unbiased estimate) because there were no observed malaria events in the CTX continuation arm. Inf, infinity.

^2^Due to the low rate of malaria events in the CTX continuation arm, separate models were used to estimate the malaria IRR in each subgroup. Thus, no interaction *p*-value is given.

**Table 4:** Subgroup analyses by ART duration: comparing CTX discontinuation to CTX continuation arms.

Outcome	ART Duration ≤ 5 y	ART Duration > 5 y	*p*-Value for Interaction
Combined outcome: malaria, pneumonia, diarrhea, and mortality	2.28 (1.39, 3.75)	2.22 (1.13, 4.35)	0.94
Mortality	—	—	—
Malaria	32.19 (5.66, +Inf)^1^	10.23 (1.31, 79.58)	—^2^
Pneumonia	2.22 (0.68, 7.18)	0.34 (0.04, 3.28)	0.15
Diarrhea	1.04 (0.55, 1.97)	2.19 (0.90, 5.34)	0.18

Data given as IRR (95% CI). IRRs were estimated using Poisson regression with robust error variance, except where indicated.

^1^Estimated with exact Poisson regression (median unbiased estimate) because there were no observed malaria events in the CTX continuation arm. Inf, infinity.

^2^Due to the low rate of malaria events in the CTX continuation arm, separate models were used to estimate the malaria IRR in each subgroup. Thus, no interaction *p*-value is given.

Reported bednet use at enrollment was high, at 88%. Reported bednet use during follow-up was high (96.4%) and was similar between both arms.

There were 34 SAEs, of which 27 were grade 3 or higher AEs (including one death), and 18 were deemed potentially related to the research (five in the CTX continuation arm and 13 in the CTX discontinuation arm) (Table 2). The rate of potentially research-related SAEs was 2.0 per 100 person-years in the CTX continuation arm and 5.1 per 100 person-years in the CTX discontinuation arm, with an IRR of 2.60 (95% CI 0.93–7.28). There were a total of 413 grade 2 or higher AEs. The most frequently reported AEs were anemia (defined as hemoglobin < 10 g/dl; *n* = 108), non-malaria febrile or flu-like illness (*n* = 53), upper respiratory tract infection (*n* = 49), diarrhea (*n* = 38), and malaria (*n* = 34). Anemia was more frequent in the CTX discontinuation arm: there were 42 anemia events in the CTX continuation arm and 66 anemia events in the CTX discontinuation arm. The rates were 16.6 per 100 person-years in the CTX continuation arm and 26.1 per 100 person-years in the CTX discontinuation arm, a statistically significant difference (IRR = 1.57, 95% CI 1.08–2.29; *p* = 0.02). There were no hypersensitivity reactions.

The estimated rate of grade 2 or higher AEs was 63.5 per 100 person-years in the CTX continuation arm and 99.5 per 100 person-years in the CTX discontinuation arm, a statistically significant difference (IRR = 1.57, 95% CI 1.29–1.91; *p <* 0.001).

**Fig 3:**
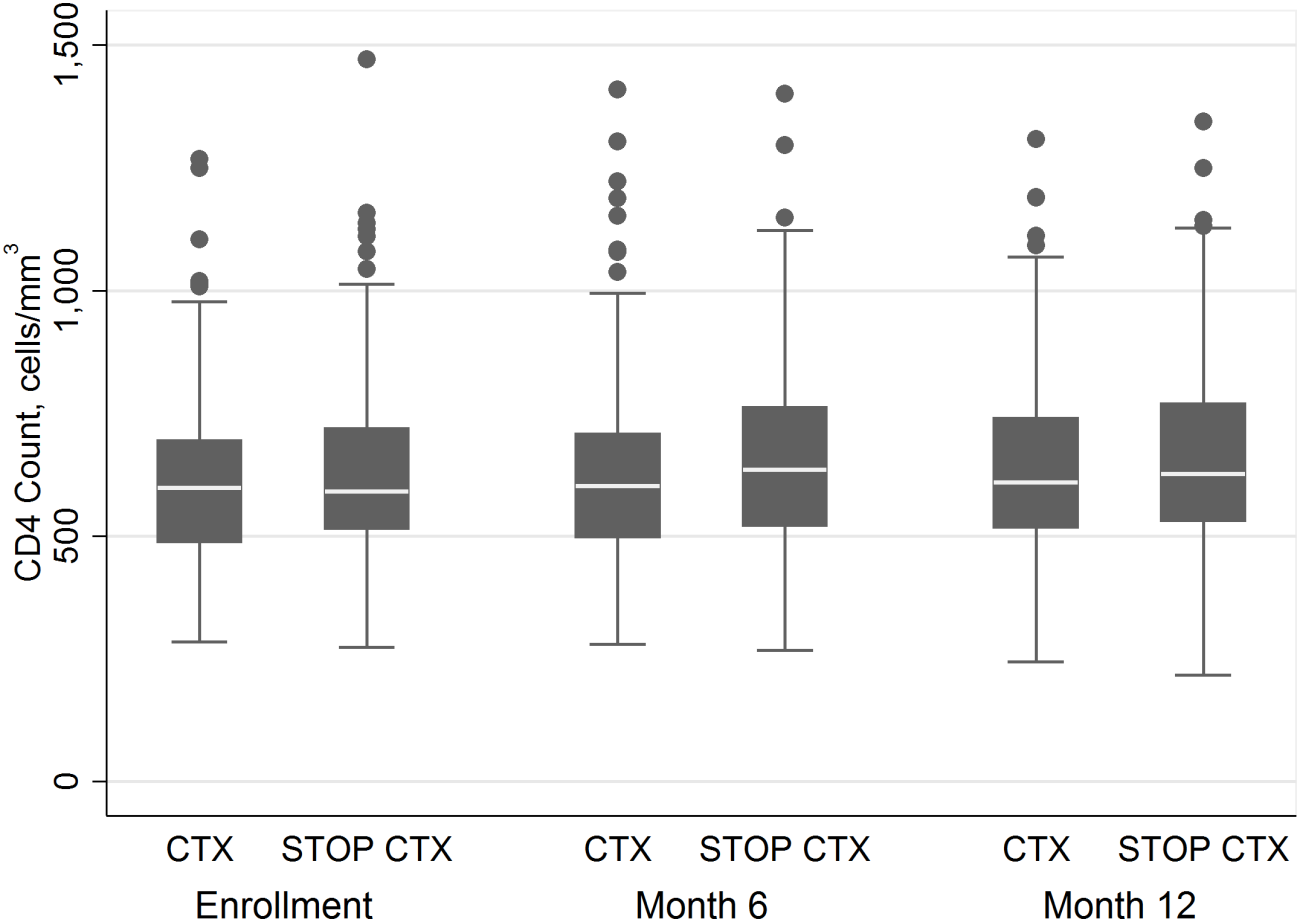
CD4 count by arm at 0, 6, and 12 mo.

Analyses of 12-mo CD4 change included CD4 counts at enrollment, at month 6, and at month 12 for all participants contributing data at these time points. There was an increase in CD4 count of 28.8 cells/mm^3^/y (95% CI 7.8–49.9) in the CTX continuation arm and of 31.6 cells/mm^3^/y (95% CI 10.7–52.6) in the CTX discontinuation arm (Fig 3). There was not a statistically significant difference in the rate of increase of CD4 count between arms (*p* = 0.85).

There were a total of nine ART treatment failure events: six immunologic treatment failures, two virologic treatment failures, and one clinical treatment failure. Five ART treatment failure events were in the CTX continuation arm, and four were in the CTX discontinuation arm. There was no statistically significant difference between arms in time to treatment failure (*p* = 0.73).

## Discussion

In this randomized trial, we compared discontinuation of CTX prophylaxis with continuation of CTX prophylaxis in HIV-1-infected adults who had evidence of immune recovery following ART. Despite having received ART for a median of more than 4 y and having immune reconstitution, adults who stopped CTX prophylaxis had significantly higher incidence of the combined morbidity/mortality endpoint, driven by malaria morbidity. Of the 34 cases of clinical malaria that occurred during our study, 33 were in the CTX discontinuation arm. Malaria cases occurred despite all study participants having received insecticide-treated bednets and a high rate of reported bednet usage (88%).

Only one other randomized control trial to date has examined CTX discontinuation after ART initiation in adults in a resource-limited setting [10]. A recent pediatric trial [9] suggested that continuing CTX prophylaxis after 96 wk of ART remained beneficial compared to stopping prophylaxis, with fewer hospitalizations for both malaria and infection not related to malaria. Similar to these studies, we found that discontinuing CTX prophylaxis in ART-treated adults resulted in an increased incidence of malaria. We did not observe a statistically increased risk of diarrhea or other infectious morbidity, but cannot include non-inferiority of CTX discontinuation for these outcomes because of low power for these comparisons.

Data from this study were presented at the Conference on Retroviruses and Opportunistic Infections in 2014 [26] and subsequently influenced, along with the study by Campbell et al. [10], the December 2014 WHO supplemental guidelines [18] as the two RCTs providing high-quality evidence that continuing CTX reduced hospitalization and malaria [26] as well as pneumonia and diarrhea [10] in settings where malaria and/or serious bacterial infections were highly prevalent.

The prior CTX discontinuation study in Uganda was stopped early after the researchers observed increased malaria, and therefore it had a limited follow-up time of up to 4 mo [10]. It is possible that the early increased rates of malaria in that trial reflected a transient “rebound” malaria and that malaria rates could have subsequently decreased as host immune responses to malaria returned. The early cessation of that study made it impossible to know whether this was indeed a transient effect. Our study, however, observed persistently increased risk of clinical malaria after CTX discontinuation during 12 mo of follow-up, without evidence of early rebound. It is also possible that the protective effect of CTX in immune-reconstituted, ART-treated individuals may be seen in HIV-uninfected individuals in malaria-endemic regions. CTX has not been similarly evaluated in HIV-uninfected individuals and has not been considered a malaria prophylaxis drug, yet it does have antimalarial activity [27]. Morbidity data for HIV-negative individuals in the Homa Bay region are limited, and we did not include a control group of HIV-1-uninfected adults for comparison. Therefore, we are unable to compare rates of malaria in our population of HIV-infected individuals to those in the community. Our study demonstrates lower malaria incidence in HIV-1-infected individuals given CTX prophylaxis. It is also possible that CTX’s effect may contribute to declines in regional malaria in settings of high HIV-1 seroprevalence with widespread CTX use. Homa Bay, for example, has an HIV-1 prevalence of close to 22% and is a region with endemic malaria [28]. HIV increases susceptibility to malaria, and with increased prevalence of untreated HIV, it is conceivable that malaria transmission could be amplified. Conversely, with the wide use of CTX in high-HIV-prevalence settings, malaria transmission could decrease.

In contrast to other CTX discontinuation studies, we did not observe an increase in diarrhea in participants in the CTX discontinuation arm [9,10]. Over 80% of participants at baseline reported boiling or purifying their drinking water. Water filter use could have contributed to this finding, as recent data suggest that provision of water filters may confer significant benefit in reducing diarrhea among HIV-infected adults [29]. In the study by Campbell et al. [10], participants used safe water vessels with chlorine solution, and use of CTX provided additional protection against diarrhea, despite high rates of enteric pathogen resistance to CTX. The etiology of diarrhea in our cohort, however, was not determined, and the incidence of diarrhea in the community is unknown. We aligned our definition of diarrhea with prior studies [10,30]; however, because pneumonia was not defined in these publications, we used a conservative, clinically relevant definition with higher severity requirements. This may have skewed case distribution, because diarrhea included mild cases, while only severe pneumonia contributed to the endpoint.

This study complements findings by Walker et al. [11], who noted a mortality benefit from CTX through 18 mo of ART, but not thereafter, and a reduced frequency of malaria throughout the follow-up period. Additionally, our data suggest that even in patients with higher CD4 counts and a longer duration on ART, CTX is still protective for malaria. For diarrhea, the effect of CTX appears protective in individuals with lower CD4 counts but not in individuals with higher CD4 counts. Overall, our cohort’s median CD4 count was high, which corresponds with the cohort’s relatively long median duration on ART (4.5 y).

Our study demonstrated low rates of treatment-limiting AEs such as hematological AEs, rash, or hypersensitivity among participants who continued CTX. All participants were on CTX at the study start, therefore likely limiting the group of participants who would have reactions to the antimicrobial and suffer from adverse effects. AEs were significantly more common among those who stopped CTX, consistent with prior studies [1–3,6,10,11].

Our study had several strengths and limitations. Because our study was an unblinded clinical trial without a placebo, clinician’s care decisions, as well as the patients’ threshold to come in for care, may have been influenced by trial arm. A second limitation, for statistical analyses, was an observed incidence of morbidity events that was lower than expected. Additionally, our study was limited to 12 mo of follow-up. A strength of the study was high retention (98% in each arm). The PSC at the Homa Bay District Hospital had a long history of collaboration with Médecins Sans Frontières and a reputation for excellent care, which contributed to retention in the trial. Participants had median CD4 counts approaching 600 cells/mm^3^ in both arms and had been on ART for a median of over 4 y with few prior hospitalizations in the past 3 mo. This, coupled with high retention, suggests that these patients may represent an elite subgroup of patients who took advantage of a strong PSC.

In conclusion, CTX discontinuation among ART-treated adults in a region with endemic malaria results in increased incidence of clinical malaria but not pneumonia or diarrhea. The implications are broad, and our results suggest that CTX prophylaxis should continue in regions with endemic malaria. However, in regions without malaria or with less endemicity, CTX cessation may be possible after immune recovery.

## Supporting Information

S1 Table Morbidity and mortality and adverse event incidence rates by study arm: per-protocol analysis. (DOCX)

S1 Text Trial protocol. (PDF)

S2 Text CONSORT statement. (DOC)

S3 Text Interim analysis plan. (DOCX)

## Abbreviations

AE: adverse event
ART: antiretroviral therapy
ARV: antiretroviral
CTX: cotrimoxazole
DSMB: data safety and monitoring board
HIV-1: human immunodeficiency virus type 1
IRR: incidence rate ratio
ITT: intention-to-treat
PP: per-protocol
PSC: Patient Support Center
RCT: randomized controlled trial
SAE: serious adverse event
WHO: World Health Organization
