# Supplementary material for: Cotrimoxazole Prophylaxis Discontinuation among Antiretroviral-Treated HIV-1-Infected Adults in Kenya: A Randomized Non-inferiority Trial
Source: PLoS Med. 2016 Jan 5;13(1):e1001934. doi: 10.1371/journal.pmed.1001934 (PMC4701407; doi:10.1371/journal.pmed.1001934)
Supplement: S1 Table — (DOCX) [file pmed.1001934.s001.docx]

S1_Table. Morbidity and mortality and adverse events incidence rates by study arm – Per-Protocol

|  | **CTX**  **Incidence per 100 person years**  **(number of cases)**  **253.5 py total** | **STOP CTX**  **Incidence per 100 person years**  **(number of cases)**  **249.2 py total** | **Incidence rate ratio (IRR)^1^**  **(95% CI)** | **p value^2^** |
| --- | --- | --- | --- | --- |
| Combined outcome: malaria, pneumonia, diarrhea, and mortality | 13.4 (34) | 30.5 (76) | 2.27 (1.52, 3.39) | < 0.001 |
| Mortality | 0.4 (1) | 0.0 (0) | 1.02 (0.00, 39.66)^3^ | 0.99 |
| Malaria | 0.4 (1) | 13.2 (33) | 33.56 (4.60, 245.01) | 0.001 |
| Pneumonia | 2.8 (7) | 3.6 (9) | 1.31 (0.49, 3.51) | 0.59 |
| Diarrhea | 9.9 (25) | 13.6 (34) | 1.38 (0.83, 2.31) | 0.21 |
| SAEs | 4.3 (11) | 8.4 (21) | 1.94 (0.94, 4.01) | 0.073 |
| Grade 3 or higher SAEs | 3.6 (9) | 6.4 (16) | 1.81 (0.80, 4.08) | 0.153 |
| SAEs deemed potentially related to the research | 2.0 (5) | 4.8 (12) | 2.44 (0.86, 6.91) | 0.093 |
| Grade 2 or higher AEs | 63.5 (161) | 98.7 (246) | 1.55 (1.27, 1.89) | <0.001 |

^1^ IRRs estimated using Poisson regression with robust error variance, except where indicated.

^2^ For test of null hypothesis: IRR=1.
