# Supplementary material for: Cotrimoxazole Prophylaxis Discontinuation among Antiretroviral-Treated HIV-1-Infected Adults in Kenya: A Randomized Non-inferiority Trial
Source: PLoS Med. 2016 Jan 5;13(1):e1001934. doi: 10.1371/journal.pmed.1001934 (PMC4701407; doi:10.1371/journal.pmed.1001934)
Supplement: S1 Text — (PDF) [file pmed.1001934.s002.pdf]

Title: Discontinuation of trimethoprim-sulfamethoxazole prophylaxis in adults on antiretroviral therapy in Kenya: a randomized trial.

PRINCIPAL INVESTIGATOR

1. **Benson Singa**: MBChB, MPH, Research Scientist, Kenya Medical Research Institute.

PROTOCOL CHAIR

2. **Christina Polyak**: MD, MPH, Visiting Scientist, Kenya Medical Research Institute. **Research Physician, Military HIV Research Program, Walter Reed Army Institute of Research** and Clinical Instructor, Department of Medicine, University of Washington.

ASSOCIATE INVESTIGATORS:

3. **Juma Rashid:** MBChB, MMed, Ag, Director, Center for Clinical Research; Chief Research Officer, KEMRI.
4. **Judd Walson**: MD, MPH, Visiting Scientist, Kenya Medical Research Institute/ Department of Medicine, University of Washington.
5. **John Waitumbi**: PhD, KEMRI-WRP, Malaria Research Laboratory, Walter Reed Project, Kisumu
6. **Barbra Richardson**: PhD, Research Associate Professor, Department of Biostatistics, University of Washington.
7. **Grace John-Stewart**: MD, PhD, Departments of Global Health, Medicine, and Epidemiology, University of Washington.
8. **Jacqueline Naulikha**: CPHN, Nyanza Coordinator, University of Washington Kenya Projects.
9. **Jacob Johnson**: PhD, Executive Officer. USAMRU-K, Nairobi, Kenya.
10. **Edwin Kamau: PhD, Director, Malaria Drug Resistance Laboratory, Walter Reed Project, Kisumu, Kenya.**

TABLE OF CONTENTS

ABSTRACT....3
LIST OF ABBREVATIONS....5
Introduction and Background....6
Objectives....7
Design and Methodology....8
Study Procedures....10
Laboratory Procedures:....13
Ethical Considerations....20
References....28

ABSTRACT

In sub-Saharan Africa, HIV continues to be a leading cause of morbidity and mortality. Approximately 22 million people are infected with HIV with 72% of AIDS-related deaths worldwide occurring in the region. For countries with high prevalence of HIV and limited health infrastructure, the WHO endorses universal TMP/SMZ for all HIV-infected individuals. Notably, these guidelines were created prior to the scale-up of ARTs. Following ART, TMP/SMZ may no longer be required. The threshold for TMP/SMZ discontinuation after ART initiation remains undefined. The effect of discontinuing TMP/SMZ prophylaxis on malaria incidence in patients on ART is not well defined. We propose a prospective randomized trial among HIV infected individuals on ART with evidence of immune recovery (ART for $\geq$ 18mo and CD4 >350 cells/mm^3) to determine whether continued TMP/SMZ prophylaxis confers benefits in decreasing morbidity (malaria, pneumonia, diarrhea), mortality, CD4 count maintenance, ART treatment failure and malaria immune responses.

HIV infected adults who have been on HAART for 18 months or more and with CD4 >350 cells/mm^3 will be consented, enrolled and randomized into continuing or stopping TMP/SMZ. Additionally, two comparison control cohorts will be established first, to determine whether TMP/SMZ modifies anti-malarial immune responses, in a cohort of recently diagnosed HIV-positive individuals who are presenting for initial HIV care, and therefore are pre-TMP/SMZ prophylaxis and ART ineligible and second, to characterize background infectious diseases morbidity in a cohort of HIV-uninfected adults in order to compare their morbidity, a proxy for baseline community morbidity, to that in individuals within the RCT cohort.

All enrolled participants will be followed up every 3 months for a period of 12 months. At each visit, participants will have clinical data collected and blood draws done for laboratory investigations including malaria tests, CD4 counts, and plasma separated for storage for immunological tests, viral loads and other future investigations.

This study will allow us to understand the effects of discontinuing TMP/SMZ on morbidity, especially related to malaria, and to compare it to baseline rates of illness crucial to safely recommending the discontinuation or providing evidence for continuation of TMPSMZ prophylaxis.

Role of Investigators:

Benson Singa:	KEMRI Principal Investigator. Responsible for study design, protocol preparation, providing oversight of the clinical management of all enrolled participants, data analysis and report preparation**. Dr. Singa is the only member of the team with access to identifiable data and a direct role in the supervision of staff.**
Christina Polyak:	Protocol Chair. Responsible for overall study design, protocol preparation, **serve as technical advisor and subject matter expert for study execution**, data analysis and manuscript preparation**. Dr. Polyak will not have access to identifiable data, will not participate in generating primary data nor will she have any interaction with study volunteers.**
Judd Walson:	Study design, protocol preparation, data analysis and report preparation.
John Waitumbi:	Study design, laboratory methods and procedures, sample testing, and analysis and quality control of laboratory samples. **Dr. Waitumbi will not have access to any identifiable data.**
Barbra Richardson	Study Statistician: Study design, randomization, and data analysis and report writing
Grace John-Stewart:	Extensively involved in the study design and the senior scientist on this protocol. Will contribute to the study implementation, analysis and reporting of results.
Jacqueline Naulikha:	Study design, field oversight, and implementation of this research.
Jacob Johnson:	Malaria diagnostics **technical expertise. Dr. Johnson will not have access to any identifiable data.**
Edwin Kamau	Malaria diagnostics, laboratory methods and procedures, analysis and quality control. Dr. Kamau will not have access to any identifiable data.

LIST OF ABBREVATIONS

TMP/SMZ	Trimethoprim/sulfamethoxazole
ART	Antiretroviral therapy
WHO	World Health Organization
ITN	Insecticide treated nets
RCT	Randomized control trial

Introduction and Background

In sub-Saharan Africa, HIV continues to be a leading cause of morbidity and mortality. Approximately 22 million people are infected with HIV with 72% of AIDS-related deaths worldwide occurring in the region. Both antiretroviral therapy (ART) and prevention of opportunistic infections have been associated with significantly decreased mortality in HIV-infected individuals. In the United States and Europe, trimethoprim-sulfamethoxazole (TMP/SMZ) prophylaxis is recommended only for HIV-infected individuals with severe immunosuppression (CD4<200 cells/mm^3) to prevent pneumocystis pneumonia and toxoplasmosis (1). In Africa, infections such as malaria, bacterial pneumonia and diarrhea remain common in both the general and HIV-infected population. Therefore, TMP/SMZ is recommended at higher CD4 counts to decrease mortality and morbidity in HIV-infected individuals (2,3,4,5). Current World Health Organization (WHO) guidelines recommend TMP/SMZ for HIV-infected individuals regardless of WHO clinical stage with CD4 counts <350 cells/mm^3 (6). For countries with high prevalence of HIV and limited health infrastructure, the WHO endorses universal TMP/SMZ for all HIV-infected individuals. Notably, these guidelines were created prior to the scale-up of ARTs.

Following ART, TMP/SMZ may no longer be required. In well-resourced settings, studies suggest very low risk of pneumocystis pneumonia after stopping TMP/SMZ prophylaxis in patients with adequate immune recovery (CD4 ≥ 200 cells/mm^3) (7, 8) and policies recommend that TMP/SMZ prophylaxis be discontinued at this point. However, the majority of studies assessing TMP/SMZ in resource-limited settings were conducted prior to the availability of ART. Therefore, the threshold for TMP/SMZ discontinuation after ART initiation remains undefined. Two studies have suggested mortality benefits of maintaining TMP/SMZ prophylaxis following ART initiation for a period of time (9, 10). These were observational studies with limitations in study design. No randomized clinical trials have assessed safety of discontinuing TMP/SMZ prophylaxis following ART immune recovery in low resource settings.

Presently, in sub-Saharan Africa, there are marked differences in national policy regarding TMP/SMZ prophylaxis in the context of ARVs with some countries such as Kenya and Uganda continuing prophylaxis indefinitely and others recommending discontinuation when patients meet certain clinical thresholds (ART use for 12 months and a CD4 > 350 cells/mm^3) (6). Current WHO guidelines on TMP/SMZ prophylaxis suggest that discontinuation be based on clinical judgment and laboratory parameters, if available. Continuing TMP/SMZ prophylaxis indefinitely raises concern about unnecessary antibiotic exposure, side effects, resistance, and pill burdens.

The effect of discontinuing TMP/SMZ prophylaxis on malaria incidence in patients on ART is not well defined. HIV disease itself impairs acquired immunity to malaria. Mermin et al. (4) showed a combination of TMP/SMZ prophylaxis and ART resulted in a 92% lower malaria rate. In another Ugandan study, patients on ART who discontinued TMP/SMZ prophylaxis had increased risk of malaria (11). However, it is not clear whether the incidence of malaria after TMP/SMZ discontinuation occurs at similar levels as the HIV-uninfected local population or if while on TMP/SMZ, the loss of natural immunity leads to a 'rebound' increase in malaria when TMP/SMZ is stopped. Moreover, it is also uncertain if these findings from Uganda would generalize to other African settings with varied malaria endemicity. In addition, use of insecticide-treated bed nets (ITNs) has been associated with marked decreases in malaria

incidence (12). It is possible that support of ITN use would attenuate malaria incidence following discontinuation of TMP/SMZ.

We propose a prospective randomized trial among HIV infected individuals on ART with evidence of immune recovery (ART for $\geq$ 18mo and CD4 >350 cells/mm^3) to determine whether continued TMP/SMZ prophylaxis confers benefits in decreasing morbidity (malaria, pneumonia, diarrhea), mortality, CD4 count maintenance, ART treatment failure and malaria immune responses. Our primary aim is to compare morbidity, mortality, and immune response to malaria in those randomized to continue versus discontinue TMP/SMZ in this randomized control trial (RCT) cohort.

For the secondary aims, two comparison control cohorts will be established. First, to determine whether TMP/SMZ modifies anti-malarial immune responses, we will recruit a cohort of recently diagnosed HIV-positive individuals who are presenting for initial HIV care, and therefore are pre-TMP/SMZ prophylaxis and ART ineligible (CD4$\geq$350 cells/mm^3) (13), and follow them for one year to compare their malaria immune responses pre TMP/SMZ and post one-year of TMP/SMZ prophylaxis. Additionally, we aim to further characterize background infectious diseases morbidity in a cohort of HIV-uninfected adults in order to compare their morbidity, a proxy for baseline community morbidity, to that in individuals within the RCT cohort.

Together, these studies allow us to understand the effects of discontinuing TMP/SMZ on morbidity, especially related to malaria, and to compare it to baseline rates of illness. Understanding the effects of discontinuing TMP/SMZ on malaria, pneumonia and diarrhea is crucial to safely recommending the discontinuation of TMPSMZ prophylaxis.

Objectives

General Objective: To determine whether continued TMP/SMZ prophylaxis confers benefits in decreasing morbidity in individuals with reconstituted immune systems.

Primary aims:
Among HIV-infected individuals who have received ART for $\geq$18 months and whose CD4 is $\geq$350 cells/mm^3, our specific aims within the randomized control trial are:

1. To compare 1 year incidence of severe infectious morbidity (malaria, pneumonia, diarrhea), mortality and malaria-immune responses in those randomized to discontinue TMP/SMZ versus continue TMP/SMZ prophylaxis. (See p.14 for outcome definitions)
Hypothesis: ART-treated immune reconstituted HIV-infected adults who stop TMP/SMZ will not have increased morbidity.

2. To compare 12 month CD4-increase in those randomized to continue vs. discontinue TMP/SMZ prophylaxis.
Hypothesis: ART-treated immune reconstituted HIV-infected adults who stop TMP/SMZ will not differ in rate of subsequent CD4 increase.

3. To compare rate of ART treatment failure in those randomized to continue vs. discontinue TMP/SMZ.
Hypothesis: ART-treated immune reconstituted HIV-infected adults who stop TMP/SMZ will not differ in rate of treatment failure.

Secondary aims:

(Comparison control cohorts as mentioned above)

a) To compare malaria immune responses before and after one year of TMP/SMZ prophylaxis, in a group of HIV-infected individuals who have been recently diagnosed with HIV and TMP/SMZ and ART ineligible.

Hypothesis: Malaria immune responses will differ before and after 1 year of TMP/SMZ prophylaxis in HIV-infected participants not on ART.

b) To determine 1 year incidence of severe infectious morbidity (malaria, pneumonia, diarrhea) in a group of HIV-negative individuals and compare to participants in the RCT.

Hypothesis: HIV negative individuals will have a similar incidence of infections as HIV-positive individuals enrolled in the RCT who discontinue TMP/SMZ.

Design and Methodology

Study Site

The proposed study sites include HIV care and treatment clinics in Western Kenya: Kisumu East District Hospital, Kisumu West District Hospital (Kombewa) and Homa-Bay District Hospital (Figure 1 below, 14). These sites have participated in research and surveillance activities by KEMRI and/or the University of Washington and Walter Reed Program-Kenya investigators over the past years. Kenya has a population of approximately 40 million with a relatively high population growth rate of 2.58%.(15). The most recent estimate of the adult prevalence of HIV in Kenya is 6.3% with a higher prevalence in Western Kenya of 13.9% (16). Western Kenya has the highest regional prevalence of both HIV and malaria in Kenya. Life expectancy is approximately 58-59 years for both men and women. Both English and Kiswahili are official languages in Kenya however; tribal languages predominate in rural areas with most people speaking their native tribal language predominantly. The Luo people live in Western Kenya along the shores of Lake Victoria and speak Luo; they make up the largest ethnic majority in the study area. Literacy rates for Kenya as of 2008 World Bank estimates are 87% of adults over 15 years old (15).

Western Kenya is a region with the potentially highest benefit from TMP/SMZ. Determining non-superiority of TMP/SMZ in these areas would provide proof of concept that could be readily extrapolated to non-malarial regions in which TMP/SMZ benefit would be expected to be lower. At baseline, these clinics routinely collect data on weight, height, tuberculosis history, history of opportunistic infections, ARV side effects, use of prophylactic medicines such as TMP/SMZ and fluconazole, ARV regimen including adherence, CD4 count, hemoglobin, ALT, recent hospitalization including duration of hospital stay and possible referrals.

Study Population and Eligibility

Individuals who are 18 years or older and are interested in study participation will be enrolled after written informed consent is obtained. Subjects must also meet the following inclusion/ exclusion criteria:

Inclusion criteria for all:

- Participants must be at least 18 years of age.
- Participants must be willing to participate and give written informed consent.

Inclusion Criteria for the randomized control trial (RCT) include criteria above as well as:

- Participants must have been on ART for $\geq$ 18 months.
- Participants must have a CD4 count of $\geq$ 350 cells/mm^3.*****
- Participants must not be suspected of ART treatment failure.
- Participants must be willing and able to return for the scheduled follow-up visits.

Inclusion criteria for the HIV-negative cohort include:

- Participants must be HIV-negative

Exclusion Criteria:

- Participants must not be pregnant at enrollment (by urine HCG testing).
- Participants must not be breastfeeding at the time of enrollment.
- If HIV-infected, participants must be on first-line ART therapy as defined by Kenyan National Guidelines

For the HIV-negative cohort: Participants must not have chronic medical problems including but not limited to diabetes mellitus, cardiovascular disease or neurologic disorders. Or have had any procedures or treatments which may decrease immunity and thus predispose to frequent infections.

*= As per inclusion criteria for the RCT, participants will have a history of a CD4 count greater than 350 at enrollment when medical records are reviewed. CD4 count will be repeated on the day of enrollment. Results may not be available on the same day. Given CD4 counts are clinically variable, clinicians will follow algorithm described in Figure 3 to evaluate patients with CD4 less than 350 on day of enrollment.

Study Design

- Non-blinded randomized clinical trial
- For secondary aims: 2 comparison control cohorts recruited in parallel

Recruitment

HIV-infected adults who meet inclusion criteria and present to the inpatient or outpatient clinic or the study sites outlined above will be recruited by local medical personnel and will be invited to join the research study.

HIV-negative adults will be invited to join the research study from referrals by neighboring HIV Voluntary Counseling and Testing (VCT) sites and from local clinics that serve the entire community. HIV-negative adults will be motivated to attend follow-up visits by understanding that their contribution and participation will be paramount to answering this important research question.

Screening, Consent, and Enrollment

For all cohorts:

HIV-infected and HIV-negative individuals interested in participating will be referred to the study. A study clinician will determine eligibility based on inclusion and exclusion criteria.

Those who meet criteria and wish to participate will be referred for potential enrollment. All invited participants will be required to sign a written informed consent prior to enrollment. Consent will be provided in Kiswahili, Kisii, Luo and English languages. All individuals will be given the opportunity to select the language to receive the informed consent. If not literate, individuals will provide a witnessed thumbprint.

Following consent, each participant will be assigned a unique study identification number (IDnum). A card detailing the IDnum and the medical personnel responsible for enrollment will be given to each participant. They will be encouraged to carry it with them to each visit. A study questionnaire assessing baseline demographic, social, and medical information will be completed by trained personnel at each site. This questionnaire will assess past medical history including recent illnesses, past hospitalizations, recent antibiotics use and adherence to ART regimen. These data will be abstracted from patient charts to assist with patient management given the study team assumes clinical care of the patient for the duration of the study.

The questionnaire will also include questions regarding use of bed nets at home, water supply, sanitation facilities, socioeconomic status, living conditions and level of education. Detailed information regarding the location of the each participant's current residence will be collected by field workers at each site. This information will be stored along with any other potential contact information to help with patient tracing should be they be lost to follow up.

RCT cohort:

Questionnaires will ask for adherence to ART regimen. Additionally, we will cull information from existing records in the HIV care and treatment clinics regarding patients' CD4 counts, ARV regimens and history of TMP/SMZ use.

Randomization

For the RCT:

After signing informed consent and being enrolled in the RCT, participants will be randomized into one of two treatment arms. The study biostatistician, Dr. Barbra Richardson, will generate block randomization code for the sites. Both the investigators and the participants will remain blinded to study arm allocation until randomization occurs. Arm 1 will receive TMP/SMZ prophylaxis as per current Kenyan guidelines. Arm 2 will stop TMP/SMZ prophylaxis at time of enrollment. If the HIV care and treatment clinic has a stock-out of TMP/SMZ, study staff will provide the participant randomized to the TMP/SMZ arm with a month's supply of TMP/SMZ and encourage them to return to their regular clinic for TMP/SMZ when available.

For Secondary Aims:

Participants will not be randomized.

Study Procedures

RCT:

Follow-up Visits

All participants in the RCT will have scheduled study visits every 3 months at the clinic which they were enrolled. Study staff will be coordinating with HIV Care and Treatment Center providers. At each follow up visit, a standardized questionnaire designed to assess any changes in socio-demographic variables or clinical history will be performed. Participants will also answer questions regarding antimicrobial use since last visit and adherence to their study drug as well as adherence to ART. A physical examination will also be performed at each visit.

Blood samples will be collected at enrollment, months 6 and 12 for measurement of CD4 count as per Kenyan National AIDS/STD Control Programme Guidelines (NASCOP) (17). Please see laboratory section for full details. As the study is being conducted within the HIV Care and Treatment clinics of local sites, TMP/SMZ will be provided by the these centers. However, should stock-outs occur, the study will maintain a supply of TMP/SMZ to dispense to participants randomized to continue TMP/SMZ.

Of note, at enrollment, pregnant or breastfeeding women will be excluded from the study. However, it is possible that women in the cohort may become pregnant during the study. Therefore, women will be asked if they may be pregnant at each scheduled follow-up visit. We will test them for pregnancy with urine pregnancy tests. Additionally, if women believe they may be pregnant at any time during the study, we will encourage them to follow up with antenatal clinics for appropriate antenatal care. Both WHO guidelines and NASCOP (17), recommend that all pregnant women with HIV, regardless of trimester, take TMP/SMZ for prophylaxis. Therefore, if pregnant, women will be removed from the RCT and re-started on TMP/SMZ. We will continue to follow these women for the duration of their 12 month enrollment.

In addition to routine scheduled visits, participants will be encouraged to return to the clinic for assessment and treatment any time they became ill between scheduled visits. To increase likelihood of compliance to this and adequate access to care, transportation reimbursements will be covered for each sick visit. Compensation will be 200 Kenyan Shillings per patient (approximately $2). This amount was agreed upon in conjunction with the district hospitals and Ministry of Health staff. At each sick visit, a standardized questionnaire will assess participants' symptoms and a clinician will perform a physical exam. Additionally, available and clinically relevant basic diagnostic tests will be performed (e.g. malaria smear, chest radiograph, stool ova and parasite exam) to assist with diagnosis as per routine clinic practice. Additionally, pertinent microbiological samples will be taken in order to better evaluate cause of illness. If further evaluation is necessary, patients will be referred for hospitalization at the nearest facility. Clinical and laboratory records from any hospitalization during participation will be reviewed. As per Kenyan National guidelines, malaria treatment is provided free of costs to all. However, given the possibility of stock-outs, the study will provide a stock of co-artem at each study site. Additionally, the study will provide a stock of antibiotics at each study site for participants who cannot otherwise afford treatment costs.

Patient Tracing

A study staff member will check in with each patient every 2 weeks by contacting each participant to assess compliance and discuss any concerns. Participants will be contacted either by telephone call or by a text message. Method of contact will depend on patients' access to a telephone with great care and emphasis being placed on patients' confidentiality. Tracking follow-up will be entered into a database. If participants do not respond to these texts in a timely fashion, pre-determined leaders in the participants' HIV community group will assist the study staff in locating the participant. If the participant decides to exit the study, reasons for doing so will be recorded on the withdrawal case report form. If the patient was randomized to stop TMP/SMZ, they will be counseled to restart and the appropriate HIV Care and Treatment clinic will be notified. If the patient is found to be ill, they will be referred to the clinic from which they were enrolled or local clinics for clinical evaluation and the study team and study clinicians will be notified.

Completion and Exit from the Study

After 12 months of follow-up, participants will have completed follow-up and will exit the cohort. If participants exit the study prior to completion, reasons for loss of follow-up will be recorded. Patients will be counseled on restarting TMP/SMZ post exiting cohort. At the next HIV care and treatment clinic visit, they will notify their providers that they have exited the study. If additional funding is available, this proposal may be modified to extend follow-up past 12 months with IRB approval. If participants elect to withdraw from the study prematurely for any reason, a withdrawal form will be completed. This form will include a section requesting permission to use any data or specimens collected prior to withdrawing.

If participants are suspected of ART treatment failure (clinical decline as evidenced by WHO Stage 3 or 4 illness (Attachment 2, attached) (18), they will be restarted on TMP/SMZ and be treated according to Kenyan guidelines. We will continue to follow the patients but they will remain on TMP/SMZ as per Kenyan guidelines. If participants' follow-up CD4 count is <350 cells/mm^3, it will be repeated in 2-4 weeks and the algorithm in Figure 3 will be followed. CD4 testing will be completed every six months as per NASCOP guidelines. Criteria will be further developed with the assistance of the expert DSMB. Of note, clinically, CD4 counts do fluctuate over time with well noted declines during acute illness episodes so each case will be reviewed and treated accordingly.

Pre-TMP/SMZ Cohort:

Procedures similar to above. Differences include:

Follow-up visits:

All participants will have study visits scheduled every 3 months at the clinic. Although patients will have by definition not met criteria for ART, they will be followed by HIV Care and Treatment clinics as per guidelines. Therefore, study staff will be coordinating with HIV Care and Treatment Centers. Standard questionnaires will be similar to the RCT but will not include questions related to the study drug. Questionnaires will ask questions related to TMP/SMZ use as this is a common antibiotic used by all.

Additionally, as in the RCT, participants will be excluded if pregnant. Participants will be asked if they may be pregnant at each scheduled follow-up visit. We will test them for pregnancy with urine pregnancy tests.

Participants will be encouraged to return to clinic for assessment and treatment at any time they become ill between scheduled visits. As with the RCT, sick visits will include transportation reimbursements. Patients will be traced but unlike in the RCT, tracing and follow up will only occur if the patients did not show up for a routinely schedule visit each 3 months. If patients develop clinical symptoms meeting WHO Stage 3 or 4 criteria or have CD4 count meeting guidelines for ART initiation, they will be evaluated by the study clinician and will be withdrawn from this cohort. We will continue to follow these patients for the duration of their follow-up. Finally, after 12 months of follow-up, participants will have completed follow-up and will exit the cohort.

HIV Negative Cohort:

Procedures: Similar to above, differences include:

Follow-up visits:

Participants will return to the clinic every 3 months for scheduled follow-up visits, as with the pre-TMP/SMZ control cohort. Questionnaires will focus on any antibiotic use, including TMP/SMZ, and illness episodes. Pregnant women will be excluded as per above. Patients will

be traced only if they do not present for their routine follow-up visits. At each scheduled follow-up, and where appropriate at sick visits, participants will be asked about risk factors for HIV and will be counseled and tested for HIV. If patient does not wish to undergo counseling and testing at the visit, they will be referred to the voluntary counseling and testing center. After 12 months of follow-up, participants will have completed follow-up and will exit the cohort.

Study Visits for all 3 Cohorts

		Enrollment (Month 0)	Month 3	Month 6	Month 9	Month 12	Sick Visits
Study Visits							
	RCT	x	x	x	x	x	x
	Pre-TMP/SMZ	x	x	x	x	x	x
	HIV Negative	x	x	x	x	x	x
Lab Visits (see tables in laboratory procedure section for details)							
	RCT	x		x		x	x
	Pre-TMP/SMZ	x		x		x	x
	HIV Negative	x				x	x

Laboratory Procedures:

Laboratory Tests

Blood collection tubes will be labeled with the IDNum, date of collection, site of collection and the initials of the enrolling medical personnel

Depending on the study visit and the cohort, between 7.5-24mL of blood (see chart below which lists amount in mL per visit) will be drawn at each visit where laboratory investigations are being conducted (enrollment, month 3, 6, 9, 12 and sick visits). The range for each cohort is as follows: RCT (1.5-24mL), pre-TMP/SMZ (1.5-24mL) and HIV-negative (9.5-10mL). This quantity of blood is being drawn for the following assays:

CD4 measurement (5mL): It is important to note HIV Care and Treatment Centers in Kenya test CD4 counts every 6 months for HIV positive patients as part of standard of care (17). We will utilize these data for enrollment, month 6 and month 12 visits where possible. If the sites' laboratory capability is compromised or laboratory testing is not completed, study staff will draw samples. CD4 measurements then will be assessed by the FACSCalibur, FACS Count or PIMA machines at the individual study sites or at the KEMRI/University of Washington Flow Laboratory at the Centre for Clinical Research in Nairobi, Kenya. Results will then be reported back to the study providers and to the HIV Care and Treatment clinic providers. As per clarification on p.8, see Figure 3 below.

Malaria RDT and/or thick/thin smears (0.5mL) will be done at the study site with quality control by Dr. John Waitumbi of the Kenyan Medical Research Unit/Walter Reed Project Malaria Lab. Smears will also be taken at month 6 and month 12.

Full blood count (FBC) with differential (2mL) will be assessed at each study site. When this is not possible, and if clinically indicated, samples will be transported to a laboratory site where this testing can be completed if possible. Given samples must be tested within 4 hours, additional sites are limited given remote location of many sites, including Homa Bay. If clinically indicated and lab resources unavailable, patients will be asked to return to clinic for repeat FBC within 4 weeks.

HIV-1 RNA Levels (5mL) will be quantified at the Kenya Medical Research Institute/Walter Reed Project or the Kenya Medical Research Institute laboratories when funding becomes available If a participant is ill and there is a concern for treatment failure, we will follow NASCOP guidelines and utilize existing HIV Care and Treatment center procedures to obtain HIV-1 RNA levels. **NASCOP and HIV Care and Treatment centers use various labs in Kenya for fee-for-service HIV-1 RNA levels. In Homa Bay, for example, the HIV Care and treatment center uses a CDC lab to complete this service. This is not part of the research protocol but part of routine care.**

Protein microarrays (1mL) and *Luminex cytokine measurements* (5mL) will be obtained from plasma of blood samples every six months. Protein microarray samples will be frozen and stored for future testing including but not limited to comparison of humoral responses to *Plasmodium falciparum* antigens using protein microarray chips. Microarray slides will be prepared and completed by Dr. John Waitumbi of KEMRI/Walter Reed Project laboratory. Results will be confirmed at the Protein Microarray Laboratory at the University of California Irvine. Additionally luminex bioassays will be used to measure cellular cytokines as markers of immune activation. These tests will be completed by the Kenyan Medical Research Institute and partners including the Walter Reed Project and Centers for Disease Control.

Filter Paper Spots – malaria testing including RT-qPCR, parasite genotyping and HRP-2 antigen detection (2mL): 1mL of EDTA blood will be blotted on multiple filter paper spots for use in RT-qPCR malaria diagnosis and parasite genotyping (DHFR and DHPS mutation profiling). One mL of EDTA blood will be frozen at -80°C and used for HRP-2 detection. These tests will be performed by Dr. John Waitumbi's lab at the KEMRI/WRP Malaria Research Lab.

Liver Panel – NASCOP policy and HIV Care and Treatment Centers routinely recommend ALT at start of ART regimen. Routine care does not include ALT unless clinically indicated. Therefore, we will collect ALT at sick or scheduled visits if clinically indicated.

**RCT Cohort**		**Enrollment**	**Month 3**	**Month 6**	**Month 9**	**Month 12**	**Sick Visit(s)**
**Lab test**	**Vol. (mL)**						
FBC with differential	2	x		x		x	x
Malaria RDT and/or Thick/Thin Smear	0.5	x		x		x	x
CD4 measurement	5	x*		x*		x*	x
HIV-1 RNA level	5	x				x	x
Liver Panel (AST/ALT)	4						x
Luminex Ctyokine Measurements	5	x		x		x	x
Filter Paper spots – malaria including: RT-qPCR, parasite genotyping, HRP-2 antigen	1.5	x	x	x	x	x	x
Microarrays	1	x		x		x	x
**Total (maximum)**	20	20	1.5	14.5	1.5	19.5	19

*note: CD4 measurements may be needed at month 6 and month 9 if not available from the HIV Care and Treatment clinics.

**Pre-TMP/SMZ Cohort**		**Enrollment**	**Month 3**	**Month 6**	**Month 9**	**Month 12**	**Sick Visit(s)**
**Lab test**	**Vol. (mL)**						
FBC with differential	2	x				x	x
Malaria RDT and/or Thick/Thin Smear	0.5	x					x
CD4 measurement	5	x*		x*		x*	x
HIV-1 RNA level	5	x				x	
Liver Panel (AST/ALT)	4						~~x~~
Luminex Ctyokine Measurements	5	x		x		x	x
Filter Paper spots – malaria including: RT-qPCR, parasite genotyping, HRP-2 antigen	1.5	x	x	x	x	x	x
Microarrays	1	x		x		x	x
**Total (maximum)**	20	20	1.5	12.5	1.5	19.5	19

* note: CD4 measurements may be needed at month 6 and month 9 if not available from the HIV Care and Treatment clinics.

**HIV-negative Cohort**		**Enrollment**	**Month 3**	**Month 6**	**Month 9**	**Month 12**	**Sick Visit(s)**
**Lab test**	**Vol. (mL)**						
FBC with differential	2	x				x	x
Malaria RDT and/or thick/thin smear	0.5	x					x
CD4 measurement	5						
HIV-1 RNA level	5						
Liver Panel (AST/ALT)	4						
Luminex Ctyokine Measurements	5	x				x	x
Filter Paper spots – malaria including: RT-qPCR, parasite genotyping, HRP-2 antigen	1.5	x				x	x
Microarrays	1	x				x	x
**Total (maximum)**	24	10				9.5	10

Following collection of blood samples at the site, the laboratory technician at each site will perform microscopy and/or a rapid diagnostic test (RDT) for malaria on all patients at enrollment to assess for asymptomatic parasitemia. Results will be recorded in the patient's study file. Additionally, RDT and/or thick/think smear will be done for all sick visits where malaria is suspected. Results from these tests will be reported to the clinician at the site within 60 minutes of collection. PCR testing results for malaria will not be real time but will be stored on a filter paper for future testing. As described above, plasma from blood samples will be frozen and stored for future testing including but not limited to comparison of humoral responses to

Plasmodium falciparum antigens using protein microarray chips. Participants will be consented for any future testing as per consent forms.

If patients require hospitalization, additional laboratory testing may be completed by hospital clinicians. We will request permission to review any hospital laboratory results.

Specimen Transport and Handling

Specimens will be transported by G4S, a security company with experience in laboratory and specimen transport. Both Drs. Walson and Singa have experience with this company. At the UW lab, blood samples will be checked to ensure they are accurately and legibly labeled with the IDnum, site of collection, date of collection and initials of the individual who collected the prepared shipment at each site. Plasma for immunologic assays, flow cytometry, and additional future testing will be shipped to Nairobi for analysis and storage. Plasma will be frozen and stored at -80C at the University of Washington freezer facility at the University of Washington/KEMRI laboratory in Nairobi. If these facilities experience break-downs, plasma will be stored with KEMRI partners in secure locations which have power back-up to ensure that sample integrity will be maintained, such as KEMRI/ Walter Reed Project, **once WRAIR IRB approval has been completed.**

Data Management and Confidentiality

Dr. Judd Walson and colleagues from the Department of Biomedical and Health Informatics at the University of Washington and KEMRI have developed several database systems for demographic and clinical information that are currently supporting both clinical trials and surveillance activities at several sites proposed in this application.

This project will be carried out at multiple clinical sites in Kenya. Please see site descriptions in section above. Clinical and laboratory data for each participant will be abstracted from routine clinical data forms into standardized project files. These data will be entered directly into a computer at several centralized sites. Information will be cross-checked for accuracy on a bi-weekly basis. All data, both hard and soft copy, will be stored in locked cabinets and with access limited to the project staff only. Participants will be identified using a unique IDnum number. The code linking the IDNum to individual identifying information will be kept in a separate secure location by the project coordinator. Clinical staff at the sites will be able to access identifying information linked to the IDNum only to match laboratory results reported by the lab for treatment of individual patients. The decision to access the confidential log will not be made by the study investigators. The IDNum will be the only identifier captured on the data collection instrument and in the computerized database. Following the completion of all data collection, the data forms will be archived. Two years following the completion of the project, identifiers will be removed from the data.

Data verification

The data management team will confirm that all forms have been properly completed at individual sites. We will have weekly conference calls to discuss quality and monitoring. Routine quality assurance will be conducted by the Local PI and the data management team to ensure that the information in the chart matches the information on the forms. In case of incomplete forms, missing information will be gathered from the participant during a follow-up visit. A data clerk will be able to hand verify that all data is completed accurately and that the

computerized data is comparable to the paper forms. Secondary verification of accuracy will be performed by the Study Coordinator and the data management team on a bi-monthly basis.

Data Analysis Plan

AIM 1:

Our main hypothesis is that ART immune restoration will be sufficient to allow safe discontinuation of TMP/SMZ prophylaxis after at least 18 months of ART with CD4>350 cells/mm^3. To assess the success of randomization, we will compare baseline characteristics between the 2 randomization groups using Chi-Square tests for categorical data and t-tests for continuous data. While mild morbidity may be improved by TMP/SMZ, policy and treatment guidelines for prophylaxis are based on severe morbidity or mortality. The study will not have power to determine mortality benefit. In US and Europe, observational studies of TMP/SMZ and trials to inform TMP/SMZ discontinuation were based solely on morbidity data (8,9).

In our study, severe morbidity will be defined as a combined outcome of malaria, pneumonia or severe diarrhea. Pneumonia will be defined as fever, cough or tachypnea and either clinically abnormal examination of the chest or evidence of infiltrate on chest radiograph significant enough to require hospitalization. Severe diarrhea will be defined as loose stools occurring more than 3 times in a 24 hour period and accompanied by >10% fluid depletion on physical exam. Severe malaria will be defined according to the Kenya Ministry of Health, National Guidelines for Diagnosis, Treatment and Prevention of Malaria (Appendix 1). Incidence of any severe infectious morbidity will be calculated for each treatment arm and compared by randomization arm using Poisson regression.

AIM 2:

We hypothesize that TMP/SMZ will not increase the rate of continued ART immune restoration in individuals following at least 6 months of ART and >350 cells/mm^3. We will compare the rates of CD4 increase in trial arms 12 months post-randomization with linear regression adjusting for baseline CD4 counts.

AIM 3:

We hypothesize that discontinuing TMP/SMZ will not increase subsequent ART failure. To compare rate of ART treatment failure, we will compare time to CD4<350 cells/mm^3 or treatment switch due to OI/CD4 criteria using Cox regression.

Secondary Aim Cohorts

Two comparison control cohorts will be established:

a) A group of recently diagnosed HIV-positive individuals who are presenting for initial HIV care and are ART ineligible (CD4>350 cells/mm^3) and therefore by definition are pre-TMP/SMZ prophylaxis initiation will be recruited. This group will be called the “pre TMP/SMZ cohort”. We will recruit 50 individuals and follow them for 12 months. After their initial visit, they will be on TMP/SMZ as per guidelines. They will have identical follow-up, lab, and tracing procedures as the RCT (see above). This will allow us to compare malaria immune responses in HIV-infected patient’s pre-TMP/SMZ prophylaxis and post- TMP/SMZ prophylaxis. We hypothesize that malaria immune responses will differ in this cohort and the RCT cohort.

b) A group of 100 HIV-uninfected clients identified at nearby HIV Voluntary Counseling and Testing (VCT) programs with the same age eligibility criteria will be enrolled for 12 month follow-up with identical follow-up, laboratory and tracking procedures as the RCT to determine incidence of infectious morbidity. This cohort will be called the “HIV negative cohort”. Morbidity

in this cohort will be compared to the individuals in the parent RCT. This HIV-negative cohort will serve as a proxy for baseline community morbidity.

Sample Size

Morbidity estimates are based on prior morbidity incidence data from ART naïve HIV-infected individuals in Nairobi, Kenya (18). Serious morbidity (pneumonia, diarrhea, hospitalization) was observed in 160/100 person years in ART-untreated HIV-1 infected women at all CD4 levels. We assumed comparable incidence assuming some morbidity decrease due to ART but some increase due to residence in Western Kenya which has higher infectious diseases prevalence. Assuming a power of 80% and alpha of 0.05, 250 individuals per arm would enable determining non-inferiority of no TMP-SMZ with a difference assumed to be clinically relevant (18).

For the comparison control studies, we will need a total of 150 participants: 100 in the HIV negative cohort and 50 in the pre-TMP/SMZ cohort.

Description	N	Comments
Randomized Control Trial	500	250 in each group
Comparison Control Cohorts		
HIV Negative	100	
Pre-TMP/SMZ	50	HIV + but pre TMP/SMZ initiation
Total	650	

Ethical Considerations

Ethical Approval

Study approvals will be obtained from the University of Washington (UW) Institutional Review Board (IRB), **Walter Reed Army Institute of Research (WRAIR), USAMRMC ORP HRPO (United States Army Materials and Research Medical Command, Office of Research Protections, Human Research Protection Office)** and the KEMRI Ethical Review Board (ERC).

Accurate and complete study records will be maintained and will be made available to representatives of the U.S. Army Medical Research and Materiel Command (USAMRMC ORP HRPO) and all other regulatory bodies as a part of their responsibility to protect human subjects in research. A copy of the approved continuing review report and the UW IRB and KEMRI ERC approval notification will be submitted to the Walter Reed Army Institute of Research (WRAIR) HSPB/IRB and USAMRMC ORP HRPO, as soon as these documents become available. After all study related activities, including data analysis are completed, a final report may be submitted to all IRBs. Any modifications and/or deviations to the research protocol will be submitted to the UW IRB, KEMRI and **WRAIR HSPB/IRB and USAMRMC ORP HRPO for review and acceptance.**

All unanticipated problems will be reported to the UW IRB, **WRAIR HSPB/IRB, USAMRMC ORP HRPO** and KEMRI ERC in writing within 10 business days of becoming aware of the event.

Recruitment, Informed Consent and Privacy/Data Confidentiality

These sections are discussed thoroughly in the following pages of the protocol:

Recruitment: p.8
Informed Consent: p.8
Privacy and Data Confidentiality: p.15

Benefits

This study has been designed to address an area of major public health significance in resource poor settings. If we are able to show that discontinuation of TMP/SMZ in ART-treated and immune-reconstituted individuals does not increase morbidity, millions of HIV-infected

individuals in resource poor settings may benefit. Additionally, by decreasing exposure of individuals to antibiotics, namely TMP-SMZ, if they are not needed, we are decreasing the chance of antibiotic resistance for the entire community and region. This is especially important in low resource settings such as Western Kenya where at baseline, antibiotic choices are severely limited. Finally, these data will allow for an estimate of pathogen prevalence in various sites and to examine cofactors associated with a variety of infections. Such data will have tremendous benefit in assisting the Kenyan Government.

Participants themselves will benefit from treatment and examination given to them and intensive laboratory monitoring. Participants will also receive free antibiotics.

Risks

Participants may experience discomfort while answering some of the questions regarding socio-economic status. If after discussing the relevance of these questions to the study, the patients continue to experience discomfort, they will be given the opportunity to skip these questions. Counseling will be offered to participants who wish to discuss discomfort they experience. This study involves blood specimen collection. The collection of these samples involves venipuncture, which may cause discomfort, pain, introduction of infection, bleeding, fainting or bruising. Precautions will be taken to avoid introduction of infection by disinfecting the site of venipuncture and using sterile equipment. The risk of bleeding and bruising will be minimized by immediate application of pressure after venipuncture. Patients will be in the sitting or supine position during blood draws to avoid injuries from fainting. Precautions will also be taken to avoid invasion of privacy of clinical patient records by a patient identification system that is separate from the patients' medical records and will be appropriately secured to maintain patient privacy.

Discontinuing TMP/SMZ in adults who have been on ART for >18 months and shown immune recovery (CD4>350 cells/mm^3, not suspected of ART treatment failure) will present a low risk of morbidity to the participants. As described in the background statement at the start of this application, TMP/SMZ has been shown to be efficacious in preventing malaria in adults, both with HIV and without (4). However, it is unreasonable to recommend a daily dose of TMP/SMZ for all adults living in all malaria endemic areas given the risks of long term TMP/SMZ use (skin rash, hematological toxicity and liver toxicity) as well as risk of development of resistance to TMP/SMZ, an important antibiotic in much of the resource-poor world. Importantly, it is not yet known if HIV-infected patients on ART who have immune recovery, will mirror baseline rates of malaria, diarrhea and respiratory disease present in the general population. Measuring baseline prevalence in local HIV-negative patients will provide a comparison group for evaluating morbidity. As outlined below, a data safety monitoring board will be monitoring patient safety and evaluating the efficacy of the intervention.

Additionally, TMP/SMZ is not without side effects such as skin rash, hematological toxicity such as neutropenia, anemia and liver toxicity. The available studies and data from programmes in low income settings report low rates of adverse reactions (6). Routine HIV care and treatment programs, participants are not monitored with laboratory markers once starting daily TMP/SMZ prophylaxis. WHO does not recommend additional laboratory monitoring for TMP/SMZ prophylaxis (6). If severe skin rashes occur, participants will be discontinued. Routine care in Kenya includes providing verbal information on potential adverse effects of TMP/SMZ and advising recipients to present to their clinics if TMP/SMZ-related adverse events are suspected. If participants randomized to stop TMP/SMZ are experiencing significantly higher morbidity, we will halt the study.

DSMB

The Data Safety Monitoring Board (DSMB) will act in an advisory capacity to the University of Washington, Department of Medicine and the Kenya Medical Research Institute (KEMRI) to monitor patient safety and evaluate the efficacy of the intervention. The DSMB is currently being formed and a full list of members will be provided once complete.

Following initiation of enrollment and at 6 monthly intervals during the course of the trial, the DSMB's responsibilities will be to:

- Review the research protocol, informed consent documents and plans for data safety and monitoring;
- Evaluate the progress of the trial, including adherence to protocol, periodic assessments of data quality and timeliness, participant recruitment, accrual and retention, participant risk versus benefit, performance of the trial site, and other factors that can affect study outcome;
- Consider factors external to the study when relevant information becomes available, such as scientific or therapeutic developments that may have an impact on the safety of the participants or the ethics of the trial;
- Review clinical center performance, make recommendations and assist in the resolution of problems reported by the PI;
- Protect the safety of the study participants/review interim or cumulative data for evidence of study-related adverse events;
- Report on the safety and progress of the trial;

In addition, at initiation of the trial, the DSMB will determine threshold values for the interim analysis of pooled data. This predetermined threshold will be discussed with the study statistician prior to starting the study.

The DSMB may conclude each review with recommendations to continue the trial without change, modify the trial, or terminate the trial, based on pre-defined criteria established at the beginning of the trial. Recommendations for modification of the design and conduct of the trial may include:

- Modifications of the study protocol based upon the review of the safety data;
- Suspension or early termination of the study because of serious concerns about subjects' safety, inadequate performance or rate of enrollment;
- Suspension or early termination of the study because study objectives have been obtained according to pre-established statistical guidelines;
- Optional approaches for trial site and investigators to consider when the DSMB determines that the incidence of primary study outcomes is substantially less than expected, such as recommendations to increase the number of trial centers or extend the recruitment period/follow-up period; and,
- Corrective actions regarding the trial site when performance appears unsatisfactory.

Confidentiality will be maintained during all phases of DSMB review and deliberations. Only voting members of the DSMB will have access to interim analyses of outcome data by treatment group. Exceptions may be made when the DSMB deems it appropriate. DSMB members must maintain strict confidentiality concerning all privileged trial results ever provided to them. The DSMB will review data only by study group.

Dissemination of Study Results

Data will be cleaned throughout and preliminary study results will be made available within twelve months of the completion of the study.

Sponsorship:

Study will be sponsored by the Infectious Diseases Society of America (IDSA) and Department of Defense's Global Emerging Infections System (DoD-GEIS).

Figure 1. Map of Western Kenya (13)

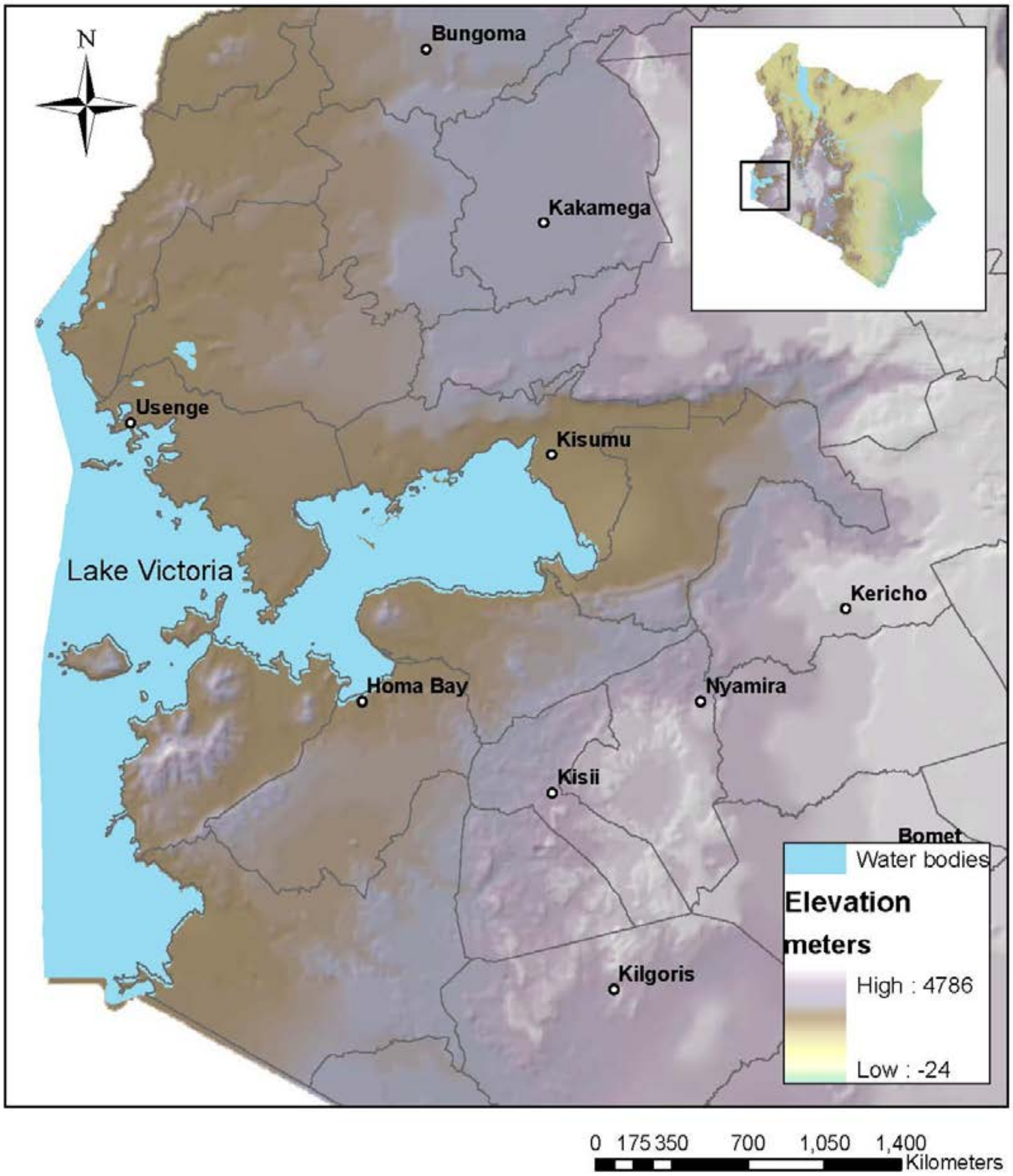

Figure 2. REVISED WHO CLINICAL STAGING OF HIV/AIDS FOR ADULTS AND ADOLESCENTS (19)

Primary HIV infection

Asymptomatic
Acute retroviral syndrome

Clinical stage 1

Asymptomatic
Persistent generalized lymphadenopathy (PGL)

Clinical stage 2

Moderate unexplained weight loss (<10% of presumed or measured body weight)
Recurrent respiratory tract infections (RTIs, sinusitis, bronchitis, otitis media, pharyngitis)
Herpes zoster
Angular cheilitis
Recurrent oral ulcerations
Papular pruritic eruptions
Seborrhoeic dermatitis
Fungal nail infections of fingers

Clinical stage 3

Conditions where a presumptive diagnosis can be made on the basis of clinical signs or simple investigations

Severe weight loss (>10% of presumed or measured body weight)
Unexplained chronic diarrhoea for longer than one month
Unexplained persistent fever (intermittent or constant for longer than one month)
Oral candidiasis
Oral hairy leukoplakia
Pulmonary tuberculosis (TB) diagnosed in last two years
Severe presumed bacterial infections (e.g. pneumonia, empyema, pyomyositis, bone or Joint infection, meningitis, bacteraemia)
Acute necrotizing ulcerative stomatitis, gingivitis or periodontitis

Conditions where confirmatory diagnostic testing is necessary

Unexplained anaemia (< 8 g/dl), and or neutropenia (<500/mm3) and or thrombocytopenia (<50 000/ mm3) for more than one month
All clinical events or conditions referred to are described in the Annexes. The UN defines adolescents as persons aged 10−19 years but, in the present document, the category of adults and adolescents comprises people aged 15 years and over for surveillance purposes.

Clinical stage 4

Conditions where a presumptive diagnosis can be made on the basis of clinical signs or simple investigations

HIV wasting syndrome
Pneumocystis pneumonia
Recurrent severe or radiological bacterial pneumonia
Chronic herpes simplex infection (orolabial, genital or anorectal of more than one month's duration)

Oesophageal candidiasis
Extrapulmonary TB
Kaposi's sarcoma
Central nervous system (CNS) toxoplasmosis
HIV encephalopathy

Conditions where confirmatory diagnostic testing is necessary:

Extrapulmonary cryptococcosis including meningitis
Disseminated non-tuberculous mycobacteria infection
Progressive multifocal leukoencephalopathy (PML)
Candida of trachea, bronchi or lungs
Cryptosporidiosis
Isosporiasis
Visceral herpes simplex infection
Cytomegalovirus (CMV) infection (retinitis or of an organ other than liver, spleen or lymph nodes)
Any disseminated mycosis (e.g. histoplasmosis, coccidiomycosis, penicilliosis)
Recurrent non-typhoidal salmonella septicaemia
Lymphoma (cerebral or B cell non-Hodgkin)
Invasive cervical carcinoma
Visceral leishmaniasis

Figure 3. KEMRI/UW CTX Study: RCT Cohort

Protocol Clarification: What do to when CD4 is <350 cells/mm3 at enrollment or follow-up visit

CD4 <350 at any visit including at enrollment

Record date and value in lab results, review with CO/SC/PI

↓

Recheck CD4 within 2 to 4 weeks

(Should NOT be rechecked during an episode of illness (e.g. fever). Defer additional 2 weeks if febrile or ill.)

↓

Recheck CD4 <350

Record second date and second value in lab results

↓

Use NASCOP 2011 criteria for immunologic treatment failure to ensure patient is not failing:*

Immunologic treatment failure is (any of the following):

- >30% drop from treatment CD4 peak OR
- Falls to or below pre-ART level
- Persistent CD4 <100 (define persistent as 2 measurements)

*note, this does not include clinical failure (based on WHO staging) or virologic failure (based on VL)

↓

CD4 <350 **AND** immunologic treatment failure:

- **Discuss with SC, MO and PIs**
- **EXIT randomization arm**
- **Restart CTX if in STOP CTX arm;** Continue CTX if in CTX arm
- Complete follow-up form during visit and endpoint
- Complete exit randomization arm form
- Manage treatment failure per clinic guidelines

EXIT Randomization Arm
CONTINUE in Study

↓

CD4 <350 but NO immunologic treatment failure

CONTINUE in Randomization Arm
CONTINUE in Study

↓

Recheck CD4 ≥350

Record second date and value in lab results

CONTINUE in Randomization Arm
CONTINUE in Study

Appendix 1 – National Guidelines for Diagnosis, Treatment and Prevention of Malaria for Health Care Workers (20).

1.2 CLINICAL FEATURES AND CLASSIFICATION OF MALARIA

The clinical course of malaria may present as uncomplicated or severe.

1.2.1 Uncomplicated malaria

This is usually characterized by fever in the presence of peripheral parasitemia. Other features may include chills, profuse sweating, muscle pains, joint pains, abdominal pain, diarrhoea, nausea, vomiting, irritability and refusal to feed. These features may occur singly or in combination

1.2.2 Severe malaria

This is a life threatening manifestation of malaria, and is defined as the detection of *p. falciparum* in the peripheral blood in the presence of any of the clinical or laboratory features (singly or in combination) listed below:

Features and definitions of severe malaria

- Prostration (inability or difficulty to sit upright, stand or walk without support in a child normally able to do so, or inability to drink in children too young to sit)
- Alteration in the level of consciousness (ranging from drowsiness to deep coma)
    - Cerebral malaria (unrousable coma not attributable to any other cause in a patient with falciparum malaria)
- Respiratory distress (acidotic breathing)
- Multiple generalized convulsions (2 or more episodes within a 24 hour period)
- Circulatory collapse (shock, septicaemia)
- Pulmonary oedema
- Abnormal bleeding (Disseminated Intravascular Coagulopathy)
- Jaundice
- Haemoglobinuria (black water fever)
- Acute renal failure – presenting as oliguria or anuria
- Severe anaemia (Hb <5g/dl or Hct < 15%)
- Hypoglycaemia (blood glucose level < 2.2.mmol/l)
- Hyperparasiteamia (parasitaemia of >200,000/µl - in high transmission area, or
- 100,000/µl in low transmission area)
- Hyperlactataemia

References

1. McNaghten AD, et al. Effects of antiretroviral therapy and opportunistic illness primary chemoprophylaxis on survival after AIDS diagnosis. AIDS 1999;13:1687-1695.
2. Anglaret X, et al. Early chemoprophylaxis with trimethoprim-sulfamethoxazole for HIV-infected adults in Abidjan, Cote d'Ivoire: a randomized trial. Cotrimo-CI Study Group. Lancet 1999;353:1463-1468.
3. Wiktor SZ, et al. Efficacy of trimethoprim-sulfamethoxazole prophylaxis to decrease morbidity and mortality in HIV-infected patients with tuberculosis in Abidjan, Cote d'Ivoire: a randomized control trial. Lancet 1999;353:1469-1475.
4. Mermin J. Effect of co-trimoxazole prophylaxis on morbidity, mortality, CD4-cell count, and viral load in HIV infection in rural Uganda. Lancet 2004;364:1428-34.
5. Hamel M, et al. Does cotrimoxazole prophylaxis for the prevention of HIV-associated opportunistic infections select for resistant pathogens in Kenyan adults? Am J Trop Med Hyg 2008;79(3):320-330.
6. World Health Organization. Guidelines on Co-Trimoxazole Prophylaxis for HIV-related infections among children, adolescents and adults. http://www.who.int/hiv/pub/guidelines/ctxguidelines.pdf
[Accessed 2 January 2010].
7. Furrer H, et al. Discontinuation of primary prophylaxis against *Pneumocystis carinii* pneumonia in HIV-infected adults treated with combination antiretroviral therapy. NEJM 1999:340(17):1301-1306.
8. Weverling GJ, et al. Discontinuation of *Pneumocystis carinii* pneumonia prophylaxis after start of highly active antiretroviral therapy in HIV infection. Lancet 1999;353:1293-98.
9. Lowrance D, et al. Lower early mortality rates among patients receiving antiretroviral treatment at clinics offering cotrimoxazole prophylaxis in Malawi. J Acquir Immune Defic Syndr 2007;46:56-61.
10. Walker AS, et al. Daily co-trimoxazole prophylaxis in severely immunosuppressed HIV-infected adults in Africa started on combination antiretroviral therapy: an observational analysis of the DART cohort. Lancet 2010;375:1278-86.
11. Campbell JD, Moore D, et al. HIV-Infected Ugandan adults taking antiretroviral therapy with CD4 counts >200 cells/uL who discontinue cotrimoxazole prophylaxis have increased risk of malaria and diarrhea. Clin Infect Dis 2012;54(8):1204-11.
12. Phillips-Howard PA, et al. Efficacy of Permethrin-treated bed nets in the prevention of mortality in young children in an area of high perennial malaria transmission in Western Kenya. Am J Trop Med Hyg 2003:68(4);23-29.
13. USAID AIDSTAR-One. Summary table of HIV treatment regimens: adult and pediatric treatment guidelines for PEPFAR focus and non-focus countries. 2009. http://www.aidstar-one.com [Accessed 2 July, 2010].
14. ESRI (2008). ArcGIS. Redlands, CA. Data from:
http://www.wri.org/publication/content/9291 [accessed 19 January, 2011].
15. The World Bank. Country Profile: Kenya.
http://data.worldbank.org/country/kenya.[Accessed 2 January, 2011].
16 USAID. HIV/AIDS Website for Kenya. http://kenya.usaid.gov/programs/health/77
[Accessed 19 January, 2011].
17. National AIDS/STD Control Programme (NASCOP). http://www.aidskenya,org/Knowledge-Centre/Publications.htm [Accessed 1 Feb 2011].
18. Piaggo G, et al. Reporting of noninferiority and equivalence randomized trials: an extension of the CONSORT statement. JAMA 2006;295(10):1152-1160.

19. World Health Organization. WHO. Interim WHO clinical staging of HIV/AIDS and HIV/AIDS case definitions for surveillance. http://www.who.int/hiv/pub/guidelines/clinicalstaging.pdf [Accessed 19 January, 2011].
20. Kenya. Ministry of Health. National Guidelines for the Diagnosis, Treatment and Prevention of Malaria for health workers in Kenya. Division of Malaria Control, Ministry of Health;2006.
