# Supplementary material for: Cotrimoxazole Prophylaxis Discontinuation among Antiretroviral-Treated HIV-1-Infected Adults in Kenya: A Randomized Non-inferiority Trial
Source: PLoS Med. 2016 Jan 5;13(1):e1001934. doi: 10.1371/journal.pmed.1001934 (PMC4701407; doi:10.1371/journal.pmed.1001934)
Supplement: S3 Text — (DOCX) [file pmed.1001934.s004.docx]

# INTERIM ANALYSIS PLAN

**Cotrimoxazole Prophylaxis Discontinuation Among Antiretroviral Treated HIV-1 Infected Adults in Kenya: a Randomized Non-Inferiority Trial**

Formal interim analyses will be conducted on Serious Adverse Events (SAEs). These will be reviewed at each DSMB meeting or earlier, if triggered by concerns of the independent safety monitor for the study. On a monthly basis, the study statistician will provide the independent safety monitor a list of all SAEs that have occurred in both study arms. The safety monitor, blinded to study arm, will decide which of these are potentially related to the research vs. clearly unrelated to the research. To increase statistical precision, SAEs deemed potentially related will be included in formal interim comparisons of SAE rates by arm and clearly unrelated SAEs will be excluded from such comparisons.

To compare SAE rates between arms, we will use Poisson regression with robust error variance, using the natural logarithm of follow-up time as an offset. We will conduct one-sided tests to monitor for harm of TMP/SMZ discontinuation, specifically a significantly higher rate of potentially related SAEs in the TMP/SMZ discontinuation arm. We will test H_0_: IRR_dc_ ≤ 1, rejecting this null if the p value is less than the alpha level designated for the test.

We will conduct these sequential analyses in accordance with the Lan-DeMets implementation of the O’Brien-Fleming boundary for harm. The overall alpha will be 0.05, and the alpha spending function will be based on an information fraction equal to (the interim number of events in all arms) / (the total expected events by the conclusion of the study). The denominator for this fraction will be forecasted using the pooled event rate observed at the time of analysis.

For safety review, we will also present results by arm for each of the study endpoints in Aims 1-4 but with no formal testing unless deemed necessary by DSMB members.
